# Identification of Alternative Allosteric Sites in Glycolytic Enzymes for Potential Use as Species-Specific Drug Targets

**DOI:** 10.3389/fmolb.2020.00088

**Published:** 2020-05-14

**Authors:** Merve Ayyildiz, Serkan Celiker, Fatih Ozhelvaci, E. Demet Akten

**Affiliations:** ^1^Graduate Program of Computational Biology and Bioinformatics, Graduate School of Science and Engineering, Kadir Has University, Istanbul, Turkey; ^2^Graduate Program of Computational Science and Engineering, Graduate School of Science and Engineering, Bogazici University, Istanbul, Turkey; ^3^Department of Bioinformatics and Genetics, Faculty of Engineering and Natural Sciences, Kadir Has University, Istanbul, Turkey

**Keywords:** allosteric regulation, glycolytic enzyme, elastic network modeling, species-specific, drug discovery

## Abstract

Three allosteric glycolytic enzymes, phosphofructokinase, glyceraldehyde-3 phosphate dehydrogenase and pyruvate kinase, associated with bacterial, parasitic and human species, were explored to identify potential allosteric sites that would be used as prime targets for species-specific drug design purposes using a newly developed approach which incorporates solvent mapping, elastic network modeling, sequence and structural alignments. The majority of binding sites detected by solvent mapping overlapped with the interface regions connecting the subunits, thus appeared as promising target sites for allosteric regulation. Each binding site was then evaluated by its ability to alter the global dynamics of the receptor defined by the percentage change in the frequencies of the lowest-frequency modes most significantly and as anticipated, the most effective ones were detected in the vicinity of the well-reported catalytic and allosteric sites. Furthermore, some of our proposed regions intersected with experimentally resolved sites which are known to be critical for activity regulation, which further validated our approach. Despite the high degree of structural conservation encountered between bacterial/parasitic and human glycolytic enzymes, the majority of the newly presented allosteric sites exhibited a low degree of sequence conservation which further increased their likelihood to be used as species-specific target regions for drug design studies.

## Introduction

Glycolysis is the most essential metabolic sequence of enzymatic reactions in all living cells that converts glucose into pyruvate to produce energy in the form of adenosine triphosphate (ATP) and reduced nicotinamide adenine dinucleotide (NADH). The process has a dual effect in the sense that while it metabolizes six-carbon sugars into smaller three-carbon compounds which are later used for a large amount of ATP production or fat synthesis, it also generates a small amount of ATP (Meyerhof and Junowicz-Kocholaty, 1943; Barnett, 2003). Thus, it is nearly ubiquitous in all living cells and essential for the survival of biological organisms. Glycolytic pathway is a sequence of ten consecutive reactions catalyzed by ten different enzymes, three of which are known to be allosteric; phosphofructokinase, glyceraldehyde-3 phosphate dehydrogenase and pyruvate kinase which appear on the third, the sixth and the last reaction, respectively.

As glycolysis is essential for living cells, allostery is equally crucial for regulating protein’s activity (Monod et al., 1963; Perutz, 1989; Koshland and Hamadani, 2002). Allostery is defined as the correspondence of conformational changes between two distant sites in the protein which usually incorporate a catalytic region and another so-called effector site. The functional state of the enzyme becomes under the regulation of a ligand or the so-called effector binding, since the catalytic region consequently becomes either accessible or inaccessible to substrates. After the first allosteric model (MWC model) proposed by Monod et al. (1965) which defined allosteric proteins as symmetric oligomers with identical protomers found in at least two conformational states (T and R) with different ligand-binding affinities (Monod et al., 1965), Weber put forward a powerful concept for allosteric regulation which is the population shift or re-distribution of protein’s conformational states (Weber, 1972). Accordingly, all proteins have a repertoire of conformational states from which they select to adopt in a particular functional state, and the ligand binding merely alters the selection of these conformations (Elber and Karplus, 1987; Pan et al., 2000). Hence, if that repertoire or the dynamic ensemble of conformations underlies the allosteric behavior, apparently one can suggest that all proteins are potentially allosteric (Gunasekaran et al., 2004). In fact, two decades old experiments demonstrated that allostery can be introduced into proteins of which their functional state do not rely on allostery, either by site-directed mutagenesis or a strong binding molecule (Falcon and Matthews, 2001; Wang and Kemp, 2001; Santamaría et al., 2002).

Allostery is merely a redistribution of conformational states as a consequence of a structural perturbation which is merely the binding of a ligand at a distal site. The same type manifestation is also recognized as a result of mutation, changes in pH, temperature, ionic strength and covalent modification such as phosphorylation and acetylation as the population shift is an intrinsically embedded dynamic feature of proteins. As previously reported for HIV protease and reverse transcriptase, the apo and ligand-bound forms of an enzyme represent two different conditions under which the receptor display distinct dynamics or communication networks (Temiz and Bahar, 2002; Kurt et al., 2003).

The general acceptance of allostery as an intrinsic feature of all proteins revolutionized the drug design efforts in an unprecedented way (Ellis, 1998; Christopoulos, 2002). One of the major advantages of targeting allosteric sites rather than catalytic or so-called orthosteric regions was the low degree of sequence conservation which enables the design of species-specific drug molecules. The first step of allosteric drug design thus involves the identification of these distinct sites away from the catalytic region which would display a high degree of sequence variability among species. For allosteric proteins, the so-called allosteric regions are usually well-established through experimental studies, yet alternative sites might exist for the same protein which will enrich the likelihood of effective drugs with greater specificity. Furthermore, for non-allosteric proteins, these “secret” allosteric sites can be exposed and used as target in drug design studies with unprecedented success.

Several well-established approaches exist to detect alternative allosteric sites. Some relies on static structures of proteins acquired from NMR or X-ray experimental studies, while others investigate large scale motions such as hinge bending via normal mode analysis (NMA) using coarse-grained elastic network model (Bahar and Rader, 2005; Tama and Brooks, 2006) or molecular dynamics simulations (Hornak et al., 2006; Lou and Cukier, 2006; Dilcan et al., 2019), since large scale motions involving large domains can be correlated with protein function. Moreover, large scale motions defined by the slowest frequency modes present an intrinsic feature of the protein (Tobi and Bahar, 2005) and also defines the distant couplings which is the nature of allostery. Therefore, it is crucial to identify potential sites in the protein that will perturb this communication and eventually the dynamic equilibrium which might lead to a functional disorder. Besides low-frequency modes, local disturbances in the conformation represented by high-frequency modes also play a critical role in transmitting signals between distant sites (Hammes-Schiffer and Benkovic, 2006; Hawkins and McLeish, 2006).

Allosteric communication in a protein is evolutionarily encoded in a protein structure and conducted via a well-defined network comprising a limited amount of conserved residues which is strongly coupled (Lockless and Ranganathan, 1999). This well-defined communication channel is evolutionized, i.e., optimized to fulfill the functional requirements with minimal energy requirement. There exist several theoretical studies which highlight the existence of functional key residues which persistently appear in pathways of allosteric signal propagation (Süel et al., 2003; Ming and Wall, 2005). Perturbations on these residues strongly affect the cooperative network within proteins and thus it is of paramount importance to develop novel approaches to effectively identify these residues. A computational study conducted by Liu and his coworkers used an ensemble-based model and suggested that functional sites may be uniquely coupled to structural fluctuations and can be identified by the way a bound ligand to these sites effect the conformational manifold (Liu et al., 2007). Another noteworthy computational algorithm developed by Flechsig makes use of *in silico* designed synthetic structures which are represented by elastic networks and a strategy of evolutionary optimization to iteratively improve allosteric coupling or signal propagation along simple pathways incorporating a set of interacting residues (Flechsig, 2017). According to the model, allostery is considered as a consequence of optimized communication between distant functional sites. Another pioneering work by Guarnera and Berezovsky emphasizes the importance of the causality and energetics of allosteric communication (Guarnera and Berezovsky, 2019). They used ligand binding and mutations as a source of perturbations and hypothesized that perturbation of functional sites can identify latent allosteric sites based on the fact that allosteric communication is symmetric in nature (Guarnera and Berezovsky, 2016a).

Our procedure in this study uses the well-known normal mode analysis using a coarse-grained elastic network model which predicts the change in the frequencies of lowest-frequency modes as a result of a ligand binding (Kaynak et al., 2018). The approach is based on the fact that as the lowest-frequency modes consist of global motions that control the protein function, the sites which would display the highest frequency shift would correspond to either active catalytic sites or potential allosteric sites. Combining this structure-based approach with an energy-based algorithm for detecting “hot spots” that are likely to be druggable sites, a powerful prediction tool was obtained. Each one of the catalytic sites was identified as strongly druggable in addition to well-recognized allosteric sites. Besides, our procedure suggested unique alternative allosteric locations observed at the interface of monomeric subunits. Interface regions in oligomeric proteins usually accommodate potential allosteric sites as the global dynamics in complex systems is most often described by the relative rearrangement of these subunits (Kurkcuoglu et al., 2011, 2015). Thus, a structural perturbation at the interface such as ligand binding most often disrupts the dynamic character and eventually the catalytic site. Moreover, proposed allosteric sites were investigated based on sequence and structural similarity between bacterial/parasitic enzyme and its human counterpart. In all these sites, a satisfactory amount of sequence variation was observed despite a high degree of structural similarity. Thus, our future drug design efforts which will target these slightly conserved sites will potentially yield species-specific drug molecules. Furthermore, our results were compared to a well-established algorithm which predict binding sites (DoGSiteScorer) using a Difference of Gaussian filter solely based on 3D structure of the protein and assess their druggability using a support vector machine which is a linear combination of three descriptors describing volume, hydrophobicity and enclosure (Volkamer et al., 2012a). The binding pockets with highest scores successfully agreed with our predictions of druggable binding sites. Despite the lack of experimental support, the observation of all well-known catalytic and allosteric sites as druggable provided a powerful critical assessment of our approach. Finally, the allosteric effect of our top druggable sites in each enzyme was confirmed via a powerful tool AlloSigMA (Guarnera and Berezovsky, 2016b; Guarnera et al., 2017), which demonstrated a decrease in the dynamics of several catalytic regions as a result of a ligand binding.

## Materials and Methods

### System Preparation

Several X-ray crystallographic structures deposited at the Protein Data Bank for three glycolytic enzymes phosphofructokinase (PFK), glyceraldehyde-3-phosphate dehydrogenase (GAPDH) and pyruvate kinase (PK) were extracted for species of Homo sapiens (*H. sapiens*) (Kung et al., 2012; Kloos et al., 2015; White et al., 2015), Staphylococcus aureus (*S. aureus*) (Mukherjee et al., 2010; Axerio-Cilies et al., 2012; Tian et al., 2018) and three parasites, Trypanosoma cruzi for GADPH (*T. cruzi*) (Guido et al., 2009), Trypanosoma brucei (*T. brucei*) for PFK (McNae et al., 2009) and Leishmania mexicana (*L. mexicana*) for PK (Rigden et al., 1999) and the selected ones were listed in Table 1 along with the details of each structure provided at the footnote section.

**TABLE 1:** Tetrameric structures of glycolytic enzymes extracted from PDB databank.

	Human	Bacterium	
Enzyme	(*H. sapiens*)	(*S. aureus*)	Parasite
PFK	4RH3^a^	5XZ7^b^	3F5M^c^ (*T. brucei*)
GAPDH	4WNI^d^	3HQ4^e^	3DMT^f^ (*Cruzi*)
PK	4G1N^g^	3T0T^h^	1PKL^i^ (*L. mexicana*)

*^*a*^4RH3; the structure with the least amount of missing residues with a resolution of 3.02 Å.^*b*^5XZ7; the only available X-ray structure reported so far with a resolution of 1.6 Å.^*c*^3F5M; the ATP-bound form of PFK enzyme with a resolution of 2.7 Å^*d*^4WNI; the T229K mutant form at 2.3 Å resolution.^*e*^3HQ4; the C151S mutant of GADPH1 complexed with NAD from S. aureus MRSA252.^*f*^3DMT; the glycosomal GADPH at 2.3 Å resolution in complex with irreversible iodoacetate inhibitor.^*g*^4G1N; M2 isoform in complex with an activator and at 2.3 Å resolution.^*h*^3T0T; MRSA PK structure at 3.1 Å resolution in complex with an inhibitor.^*i*^1PKL: the structure of Leishmania Pyruvate Kinase at 2.35 Å resolution.*

### Sequence and Structural Alignment

To identify similarities and differences between human and bacterial/parasitic species at the level of primary structure, pairwise amino acid sequence alignment was performed using Needleman-Wunsch global alignment algorithm (Needleman and Wunsch, 1970) via EMBOSS-Needle (Rice et al., 2000) web server using the following default parameters; Blosum62 as similarity matrix (Henikoff and Henikoff, 1992), gap penalty as 10 for opening and 0.5 for extension, and no end gap penalty. As for displaying the structural differences, the *super* module of PyMOL graphics visualization tool was used (Schrödinger, 2015). *Super* module superposes two structures based on the positions of backbone α-Carbon atoms regardless of their amino acid identity. It uses a dynamic programming algorithm which incorporates a series of refinement cycles to eliminate unfit pairing and thus minimizing the root mean square deviation (RMSD) between two aligned structures. Finally, each receptor structure was colored based on sequence identity, similarity and differences as well as RMSD value, to identify variations emerging at both primary and tertiary level.

### Computational Solvent Mapping (CS-Map)

Computational solvent-mapping was used to identify all possible ligand binding sites via docking small drug-like organic molecules over the entire receptor surface. For that purpose, the widely used FTMap (Brenke et al., 2009; Kozakov et al., 2015) tool was employed. As for all CS-Map algorithms, FTMap was constructed based on the assumption that binding pockets incorporating the “hot spots” provide major contributions to the free energy of binding, and thus are likely to bind drug-like ligands with high affinity (DeLano, 2002; Ciulli et al., 2006; Metz et al., 2012; Hall et al., 2015). The algorithm uses fast Fourier transform (FFT) correlation approach which effectively and quickly samples billions of probe’s poses and calculate their energies based on a detailed energy function which is CHARMM27 (Brooks et al., 1983). A total of sixteen organic probe molecules (isopropanol, acetaldehyde, phenol, benzaldehyde, urea, dimethyl ether, acetonitrile, ethane, acetamide, benzene, methylamine, cyclohexane, ethanol, N,N-dimetylformamide, isobutanol and acetone) varying in size and chemical compositions were used for docking. Initially, each probe was docked using rigid body algorithm, and a total of 2,000 generated poses were energy-minimized and clustered based on proximity. Clusters were then ranked by their Boltzmann-averaged energy values. Overlapping clusters of different probe types were assembled into consensus sites (CS) identified as “hot spots.” When several CS were found to be near each other on the surface of the protein, there is a strong indication of a potential “druggable” binding region. In a sense, FTMap mimics the experimental NMR or X-ray crystallographic studies which attempt to solve the protein structure using a variety of organic solvents which often form clusters in active sites of the protein.

In addition to solvent-mapping the overall tetrameric structure, each monomeric subunit was solvent-mapped individually. This approach increases the number of alternative solutions by enabling regions that would not be accessible in a tetrameric arrangement. Considering the fact that an X-ray structure only represents an instantaneous state of the receptor in time, the monomeric decomposition and mapping approach attempts to alleviate that drawback, and provides alternative binding sites that would not be detected otherwise. However, this approach may give rise to clusters that would be inaccessible from outside if they happen to be located at the interface of monomeric subunits and thus should be discarded.

**FIGURE 1:**
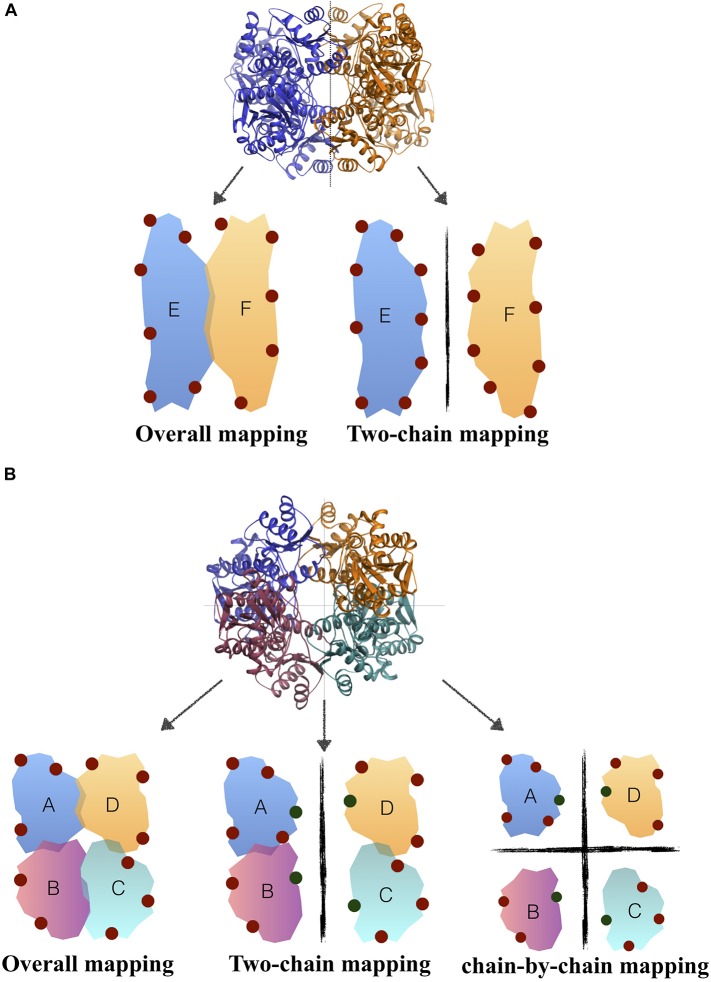
Solvent mapping strategy in **(A)** *H. sapiens* PFK, **(B)** *T. brucei/S. aureus* PFK where binding sites proposed by FTMap were illustrated with circles.

While all parasitic/bacterial species of PFK are tetrameric structures, *H. sapiens* PFK is dimeric where each monomer consists of two domains. As depicted in Figure 1A, each domain is the counterpart of one chain in tetrameric structure of parasitic/bacterial PFK. Thus, when *H. sapiens* PFK was decomposed into its monomeric subunits for solvent-mapping, bacterial PFK was also decomposed into its two-chain units corresponding to one monomeric unit in human and then solvent-mapped for compatibility, in addition to chain-by-chain decomposition. For GADPH and PK, two-chain solvent mapping was not necessary, as they were tetrameric in all species (see Figure 1B).

### ENM-Based Residue Scanning

Elastic network model (ENM) is a powerful theoretical approach used to predict the global or essential dynamics of biomolecular structures which is then used to establish the relationship between the structure and the functional mechanism (Tirion, 1996; Haliloglu et al., 1997; Doruker et al., 2000; Atilgan et al., 2001). In this model, the protein was represented as a collection of beads connected by Hookean springs corresponding to a collection of atoms connected by fluctuating bonds. Furthermore, the springs connected the atoms only if they were closer than a predefined cutoff distance of 15 Å in the native structure. In our study, we used a residue scanning method that was developed based on this coarse-grained standard ENM (Kurkcuoglu et al., 2015). In this new approach, each residue represented by its backbone α-Carbon as a single node was redefined such that side-chain heavy atoms will be included as extra nodes. It was proposed that these new additions will mimic the presence of a bound ligand interacting with that residue. The effect was then quantified by the change in the *ith* collective mode’s eigenvalue λ_*i*_ upon adding the extra nodes to the selected residue,

$$\%\mathrm{shift}\mathrm{for}\mathrm{mode}i\left(\%s_{i}\right)=\frac{\lambda_{i}\,\left(\mathrm{modified}\right)-\lambda_{i}\,\left(\mathrm{original}\right)}{\lambda_{i}\,\left(\mathrm{original}\right)}\times 100$$

The percentage shift for each residue was determined as an average over the 20 slowest modes as 20 slowest essential modes dominated more than 90% of the global dynamics of all three receptors. The average value $\%s=\sum_{i=1}^{20}\left(\%s_{i}\right)/20$ was then represented using a color gradient on the protein’s X-ray structure. The regions which incorporate residues with highest %*s*_*i*_ values were simply proposed as potential allosteric sites. Furthermore, another theoretical method DoGSiteScorer (Volkamer et al., 2012b) incorporating physicochemical pocket features and perturbation based on normal-mode analysis (NMA) has been employed to support our findings.

### Merging FTMap and ENM-Based Residue Scanning Results

Clusters identified from FTMap were further explored to identify all proximal residues situated within 5 Å of the bound solvent molecule observed in that cluster. Then, a mean percentage frequency shift value for each cluster was determined as the average over all *n* residues neighboring all the bound solvent molecules in that cluster as $\hat{S}=\sum_{j=1}^{n}\left(\%s_{j}\right)/n$. If a cluster’s $\hat{S}$ value was smaller than 50%, that cluster was simply discarded from analysis as its interaction with a ligand would have a negligible impact on the global dynamics of the receptor. In case the number of alternative solutions is scarce, the threshold value was decreased to 25%.

### Determination of Interface Regions Using Relative Solvent Accessible Surface Area (rSASA)

Interface regions are known to incorporate conserved “hot spot” residues which majorly contribute to the free energy of binding to another subunit or partner protein, thus are frequently targeted in species-specific drug design studies (Clackson and Wells, 1995; Bogan and Thorn, 1998). In addition, binding of a ligand at the interface is suggested to disrupt protein’s global dynamics which is most often governed by the close correspondence between monomeric units. In this study, the interface regions were determined based on relative solvent accessibility surface area (rSASA) which is a widely used metric to identify buried and exposed residues in the structure. rSASA was defined as a residue’s solvent accessibility (ASA) normalized by its maximum ASA value. Maximum ASA for each residue X previously reported in Tien’s work (Tien et al., 2013) was derived as the highest ASA achieved in a Gly-X-Gly tripeptide construction evaluated for all sterically possible conformations. Accordingly, a residue was found at the interface if its rASA value in the monomeric form is greater than its rASA value in the complex form (Levy, 2010).

## Results and Discussion

### Solvent Mapping and ENM Analysis Detected Several Druggable and Potential Allosteric Sites At/Near Interface Regions

Consensus sites (CS sites) or hot spots were determined for the overall tetramer, and also for each chain separately, to increase the number of alternative binding sites. In addition, for PFK enzyme only, solvent mapping was also employed on an assembly of two chains, as the corresponding PFK structure in human species existed as a dimer with each monomeric unit corresponding to two chains in bacterial/parasitic species tetrameric structure (see Figure 1A in Materials and Methods section). As listed on the third column of Table 2, for tetramer mapping, the highest number of CS sites was 18 in *S. aureus* of PK (*Sa*PK), and the lowest number was 8 observed in human GADPH (*h*GADPH). The number of CS sites in chain-by-chain mapping was comparable to that found in tetramer mapping. Overall, GADPH demonstrated the lowest amount of CS sites in all three species.

**TABLE 2:** Number of clusters determined before and after filtering protocols for three glycolytic enzymes (PFK, GADPH, and PK) in different species (*H. sapiens*, *S. aureus*, *T. brucei*, *T. cruzi*, and *L. mexicana*).

		(1) Total Number of Clusters/							
		(2) Non-Overlapping Solvent-Accessible Clusters/							
		(3) After ENM filtering (frequency shift > 25%/50%)							
Enzyme	Species	Tetramer	Chain A	Chain B	Chain C	Chain D	Chain AB*	Chain CD*	TOTAL
PFK	*H. sapiens*	13	11	12	13	9	10	11	79
		13	9	11	12	8	2	2	66
		13/13	7/3	9/5	9/5	8/5	2/2	2/2	50/35
	*S. aureus*	17	12	11	11	11	11	12	85
		17	9	8	8	8	4	4	58
		17/17	5/4	4/3	4/3	5/4	4/4	4/4	43/39
	*T. brucei*	13	10	11	9	10	13	12	78
		13	10	10	9	10	4	5	61
		13/8	8/7	8/3	6/3	7/3	4/3	5/5	51/32
GADPH	*H. sapiens*	8	7	7	9	8	–	–	39
		8	6	7	9	7	–	–	37
		8/8	3/3	3/2	4/2	3/1	–	–	21/16
	*S. aureus*	14	6	9	8	7	–	–	44
		12	3	6	6	4	–	–	31
		12/12	3/3	6/6	6/5	4/3	–	–	31/29
	*T. cruzi*	15	7	9	7	9	–	–	47
		15	5	5	5	7	–	–	37
		15/2	5/5	5/2	5/2	7/7	–	–	37/18
PK	*H. sapiens*	12	12	10	10	9	–	–	53
		12	11	9	9	8	–	–	49
		12/5	9/7	8/4	7/3	8/4	–	–	44/23
	*S. aureus*	18	8	12	10	10	–	–	58
		18	8	11	9	9	–	–	55
		18/10	6/4	7/4	7/4	7/4	–	–	45/26
	*L. mexicana*	15	9	11	9	9	–	–	53
		15	6	9	5	5	–	–	40
		15/6	3/3	7/6	5/5	2/1	–	–	32/21

*Chains AB and CD correspond to one monomeric subunit in H. Sapiens (see Materials and Methods section).

Several CS sites obtained from chain-by-chain mapping had to be discarded as they either coincided with CS sites obtained from tetramer mapping or became solvent inaccessible in tetrameric arrangement. Numbers listed in the second row of each cell in Table 2 indicate the number of non-overlapping and solvent accessible clusters. Lastly, each site in the remaining list was evaluated based on its percent frequency shift value averaged for all the residues in the immediate vicinity (%*s*), as mentioned in Materials and Methods section. Accordingly, CS sites displaying an average %*s* lower than 50% was eliminated in the first run. To increase the number of alternative binding sites, a second threshold of 25% was also used in case the number of solutions is limited. As listed in the third row of each cell in Table 2, the total number of CS sites was found to be significantly higher in PFK enzyme for all three species than either GADPH or PK. Another unexpected outcome was *S. aureus* displaying the highest number of hot spots among species for all three enzymes, with the highest number being 39 observed for PFK and all satisfying 50% frequency threshold.

The location of all consensus sites listed in Table 2 was presented extensively in Supplementary Figures S1–S3 for all three enzymes. It was noticeable that the majority of CS sites was detected at/near interface regions as indicated in blue color. Furthermore, the existence of more than one CS site situated nearby further emphasized the existence of a druggable site. Supplementary Tables S1–S3 list all these druggable sites with two or more CS sites. Some of these clusters were marked with either a single or a double star which indicate those that did not fulfill 25 and 50% frequency shift criterion, i.e., ineffective sites. CS sites with double stars were those having a frequency shift between 25 and 50%, and were used in case of limited number of alternative solutions. Isolated CS sites were those with no close proximity to any other CS sites. They were only observed for PFK and PK enzymes and listed separately in the footnote section of the corresponding table.

**FIGURE 2:**
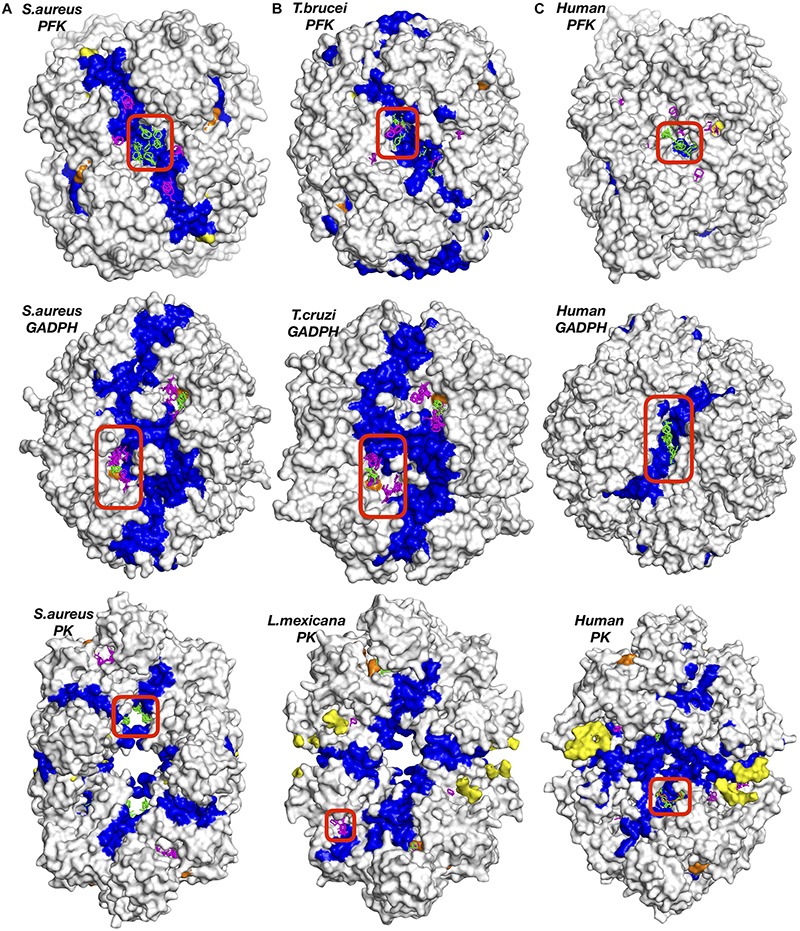
Potential druggable sites proposed for three enzymes of different species, **(A)** bacteria (*S. aureus*), **(B)** parasite (*T. brucei*, *Cruzi*, or *L. mexicana*) and **(C)** human (*H. sapiens*) using solvent mapping (FTMap). Interface regions between subunits indicated in blue color. Experimentally reported allosteric and catalytic regions were highlighted in yellow and orange, respectively. Clusters colored in green and magenta correspond to results for tetrameric and chain-by-chain solvent mapping, respectively.

To further highlight the most probable target regions, all druggable sites with two or more effective CS which gave rise to a frequency shift above 50% in global dynamics were listed in the following Table 3. In addition, residues constituting each site were determined based on their proximity to the clusters (<5Å) and listed in Supplementary Tables S4–S6 for each enzyme separately. The top druggable site on the list given in bold character incorporates the highest amount of CS and was illustrated in Figure 2 for each three enzymes of each species. PFK exhibited the highest amount of distinct druggable sites among three enzymes, varying from 5 for *S. aureus* up to 11 for *H. sapiens*. In GADPH, 2 –5 druggable sites were detected only, yet each site was crowded with several CS. Pyruvate kinase displayed a total of 5–6 druggable sites for each species, each holding 2–4 effective consensus sites. It is important to note that all druggable sites shown in Figure 2 also have symmetric counterparts which are shown in detail in Supplementary Figures S1–S3.

**TABLE 3:** Druggable sites incorporating several consensus sites labeled with an ID composed of a number and a letter.

Enzyme	Druggable site ID	*S. aureus*	Parasite	*H. sapiens*
PFK	1	4-5-7-8-12-15-17	1-9-13-6AB-7AB	4-7-8-11CD
	2	1-2-3-6-11-16	7-11-5CD-12CD	5D-6D-7D
	3	2A-10A-11A#	5-6CD-9CD-11CD	6B-7AB-1
	4	2D-9D-11D	2A-3A-5A-8A	3-5-9
	5	2B-10B	3-12-13AB	5B-10B
	6		6A-7A	4C-8C
	7		10B-11B	6-11
	8		7D-10D	10-12
	9			4B-9B
	10			6C-9C
	11			2-5C
GADPH	1	1A-2A-5A-7A-8A-9A-2	1D-3D-4D-6D-7D-9D-3	1-2-3-4-5-6
	2	2B-3B-4B-7B-8B-5	2A-3A-4A-5A-7A-12	2A-4A-7
	3	3C-4C-6C-7-8-12	1B-6B-7B	2B-4B
	4	3D-4D-5D-3-14	2C-4C	2C-5C
	5	1-4-6-10-11		2D-8
PK	1	2-3-16-18	4C-5C-6C	2-4-5-6
	2	4-5-13-15	1B-9B-11B	3A-8A-9A-12A
	3	1A-2A-6A	9C-5-14	1B-9B
	4	1B-2B-8B	8-8A	1C-5C
	5	2C-3C-5C	7-13	7D-9D
	6	2D-3D-4D		

**# The ID is composed of a single number which is sometimes followed by a letter. The number indicates the rank of that cluster; the smaller the number, the most populated the consensus site is, which increases the likelihood of that cluster. The letter indicates the mapping type, i.e., “A” indicates chain-by-chain mapping result coming from chain A, etc.**

### *S. aureus* Phosphofructokinase (*Sa*PFK) Indicated an Alternative Allosteric Region in Addition to Well-Known Allosteric and Catalytic Regions

For phosphofructokinase enzyme, all druggable sites listed in Table 3 were observed at the interface region as depicted in detail in Supplementary Figure S1. Seven CS located at the top druggable site were picked up from solvent mapping of the tetrameric structure, as illustrated in green at the top left figure of Figure 2. In the vicinity of this region, there exist isolated consensus sites obtained from chain-by-chain solvent mapping and are distinguishable by their magenta color, reinforcing the promise of this site for allosteric regulation. The second top druggable site on the list with six CS is the symmetric counterpart of the first and is located on the exact opposite face of the enzyme (see Supplementary Figure S1A). Either one of these sites can be safely proposed as an allosteric target region. Furthermore, a computational study conducted by Mitternacht et al. recognized the same exact region via Monte Carlo simulations as a possible binding site as it showed characteristics of being coupled to the intrinsic motion of the protein (Mitternacht and Berezovsky, 2011). Furthermore, this proposed region has an equivalence in human species which also incorporates top druggable site as depicted in Figure 2 (top right corner). On the other hand, as the human PFK is composed of two dimers where each monomeric unit is equivalent to two dimers in bacterial PFK, there is no interface in this proposed allosteric site. Recently, drug discovery studies aim the interface regions for identifying new allosteric drug candidates that would likely inhibit enzymatic activity through changing the global dynamics and thus preventing large dynamics subunit motion required for forming the active site (Pommier and Cherfils, 2005; Rahimova et al., 2018). Hence, the absence of any interface at the correspondin garea in human PFK might offer some advantage when designing drug molecules specific for *S. aureus* PFK.

**FIGURE 3:**
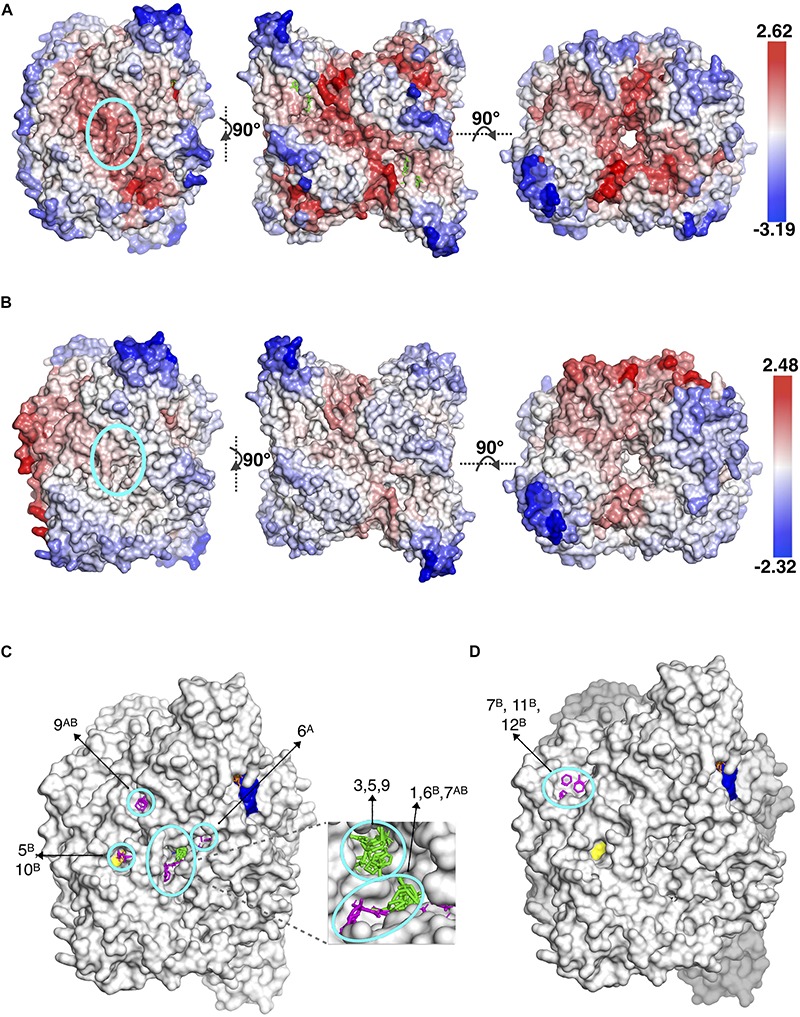
Different views of the same snapshot of human PFK colored based on frequency shift in **(A)** dimeric (inactive) and **(B)** tetrameric (active) forms, for which the top druggable site is highlighted with a cyan circle. Consensus sites at the top druggable site of human PFK in **(C)** dimeric (inactive) and **(D)** tetrameric (active) forms. Clusters colored in green and magenta correspond to results for tetrameric and chain-by-chain solvent mapping, respectively.

Moreover, our computational approach was employed for the dimeric form of human PFK which represents the inactive state, hence our conclusion would not be complete without investigating the active state of human PFK which is a tetramer. The tetrameric active form of human PFK incorporates two dimers and as each dimer corresponds to one tetrameric structure in bacteria, the human PFK becomes the equivalence of two bacterial tetramers. Consequently, an additional solvent mapping and elastic network modeling was employed using the human tetramer and the clusters with frequency shifts above 50% were collected together with clusters obtained for the human dimer only. As indicated with a color gradient in Figures 3A,B, the intensity of the frequency shifts in human tetramer in 3b was significantly lower than those in dimer form in 3a. On the other hand, as anticipated, the highest intensity of frequency shift in tetramer form was observed at the interface region through which the second subunit bind.

The proposed druggable site encircled in the left figures clearly indicate the active tetramer form displaying a lower degree of frequency shift compared to dimer form. Consequently, the number of druggable sites which satisfied the frequency shift threshold of 50% in tetramer was significantly reduced (see Figures 3C,D). This further increased the potential of our proposed site to be the most suitable target region for designing species-specific drug molecules, as the same region in active form of human PFK would not favorably accommodate any drug molecule or if that happens, the receptor’s global dynamics would not be affected by its binding as much as its bacterial counterpart would.

**FIGURE 4:**
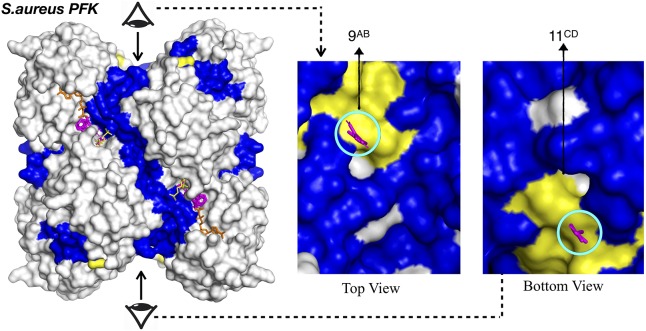
Alternative allosteric regions proposed in *S. aureus* and PFK indicated by circled consensus sites. Yellow patches indicate the experimentally observed allosteric sites (Kloos et al., 2015; Tian et al., 2018). ATP and F6P were illustrated with orange and yellow sticks. Clusters colored in magenta correspond to results for chain-by-chain solvent mapping.

The three remaining druggable sites listed for *S. aureus* in Table 3, were observed in the vicinity of the active site (depicted in orange in Supplementary Figure S1), thus they are far from functioning allosterically. Still, it clearly demonstrates the power of our computational approach to detect catalytic sites as well as allosteric sites which both require a coupling between ligand binding and protein’s intrinsic dynamics. Furthermore, there exist a second region in *Sa*PFK which incorporates one isolated CS visible at the top and its symmetric counterpart at the bottom view of the receptor as depicted in Figure 4, thus creating a region for possible allosteric regulation. No such cluster was observed in the same region in human PFK. Besides, this second alternative site is passing through an interface region, further accentuating its potential role in allostery. However, these consensus sites were detected within reach to a well-known binding site for two allosteric effectors which are the activator ADP-Mg and the inhibitor phosphoenolpyruvate (PEP), as depicted with yellow surface in Figure 4 (Schirmer and Evans, 1990). Although this site cannot be introduced as novel, the findings are supportive of our procedure’s prediction power.

**FIGURE 5:**
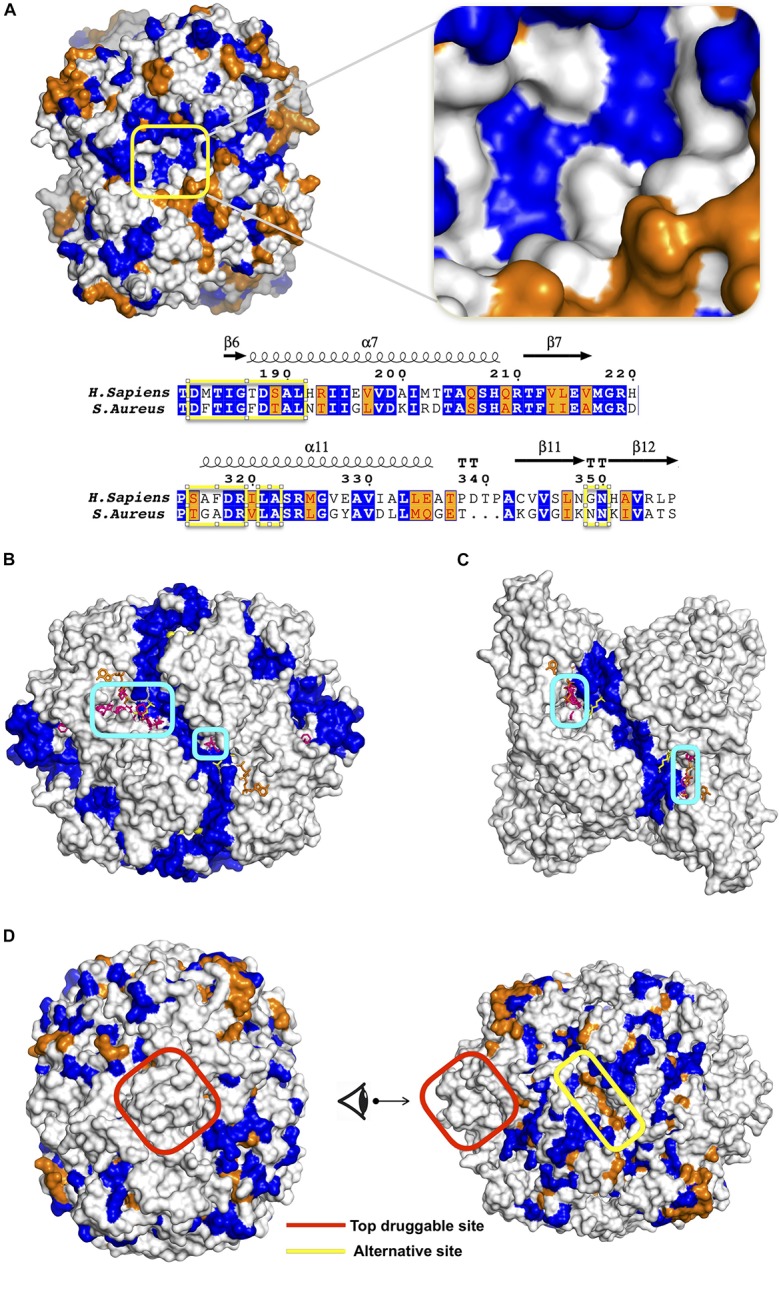
**(A)** Sequence similarity between human and *S. aureus* PFK illustrated on a snapshot and a sequence alignment with top druggable site encircled in yellow. ESPript 3.0 tool (Robert and Gouet, 2014) used for graphical illustration of sequence alignment. Potential allosteric sites represented by clusters encircled in blue for **(B)** *T. brucei* and **(C)** human PFK. ATP and F6P colored in orange and yellow, respectively. **(D)** Sequence similarity illustrated on a snapshot of *Tb*PFK at two different angles. Similar, identical and dissimilar residues colored in orange, blue and white, respectively.

For proposing a potential target region for designing species-specific drug molecules that would bind more strongly to *Sa*PFK than its human equivalence, we need to make sure that either structural or sequence conservation is minimum at the region of interest. As illustrated in Figure 5A, a snapshot of *Sa*PFK colored based on sequence similarity/identity between human and bacteria clearly displays a low degree of sequence conservation in the proposed site, as highlighted with an abundancy of white spaces corresponding to dissimilar residues. Furthermore, the overall structural RMSD between two species was determined as 1.56 Å, and this value is even lower for the confined region at the top druggable site. As the human counterpart of this proposed allosteric region in *Sa*PFK also incorporates the top druggable site with four CS as depicted in Supplementary Figure S1, the degree of variation at sequence level is satisfactorily low for proposing this site as a target in the design of species-specific drug molecules.

### *T. brucei* PFK (*Tb*PFK) Suggested an Alternative Allosteric Region in Addition to a Site Within Reach to a Catalytic Region

Similar to *S. aureus*, the top druggable site incorporating five CS was observed in a region passing through an interface and was the counterpart of the allosteric region in *S. aureus*, as illustrated in Figure 2B (top middle). In the vicinity of this region, there exist several other druggable and consensus sites strengthening its likelihood to be allosteric. Moreover, there exist two alternative sites represented by encircled areas located at close proximity to each other and to an interface region as illustrated in Figure 5B. The symmetric counterparts of these regions also exist at the opposite site of the receptor as illustrated in detail in Supplementary Figure S1. However, each of these sites coincide with the well-known binding area of the substrate F6P shown with yellow sticks, therefore unlikely to be suggested as an allosteric site. In human counterpart, a similar observation was made, i.e., two distinct druggable sites were detected as depicted in Figure 5C, each close to ATP and F6P binding area, but also near the interface region. On the other hand, the area in between these two druggable sites might be proposed as a druggable target site for allosteric drug candidates as it is passing through an interface which might perturb the global dynamics of the receptor essential for its activity. In addition, it displays a low level of sequence conservation represented by mostly white and orange spaces as in Figure 5D. Furthermore, the top druggable site displays a significantly low level of sequence conservation as depicted with a nearly white area encircled as in Figure 5D, thus would be an ideal location to be targeted for species-specific drug discovery.

### Tunnel Region Observed in GADPH Can Be a Potential Allosteric Site

**FIGURE 6:**
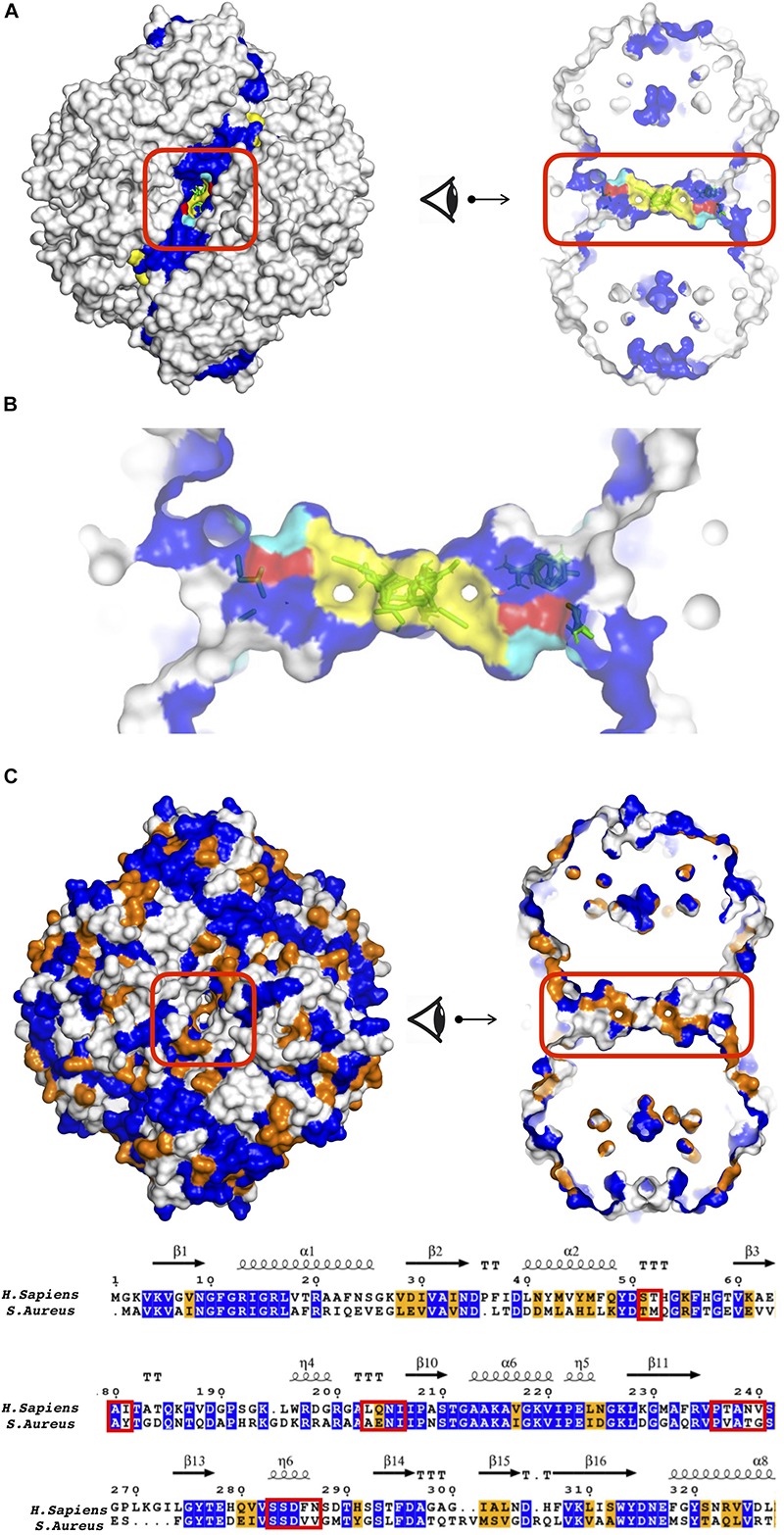
**(A)** Tunnel like region as a potential allosteric site in *S. aureus* GADPH using different perspectives. **(B)** S-loop was depicted with yellow patches, key residues S50 and S287 in the tunnel region, colored in red and cyan, respectively. Sequence similarity between *S. aureus* and *human* GADPH, illustrated on **(B)** a snapshot in two different angles and **(C)** a sequence alignment. See caption of Figure 5 for color coding. Tunnel region encircled in red.

GADPH displayed a distinct profile of druggable sites which were well packed with several CS in both bacteria and parasite. However, top sites on the list were observed near the catalytic region and thus cannot be proposed as allosteric (see Figure 2). On the other hand, our procedure accurately detects all catalytic sites in addition to allosteric ones. An additional druggable site which appeared in Table 3 with five CS for *S. aureus*, was detected in a tunnel like region passing through the center of the receptor as depicted in Figure 6A. In *T. Cruzi* GADPH, consensus sites which appeared in the same tunnel region only displayed a moderate amount of frequency shift which was determined between 25 and 50%, whereas those in human and *S. aureus* GADPH, the 50% criterion was fulfilled.

Agreeably, the tunnel like location coincided with a well-known dynamic S-loop which is known to be modulated by phosphorylation of Ser50, Ser203, and Tyr41 in regulating the enzymatic activity through NAD-binding pocket and oligomer assembly (Dubey et al., 2017). The regulatory effect of GADPH S-loop via its phosphorylation is a universal feature as the phosphorylated sites consist of well conserved residues. Dephosphorylated Ser50 and Tyr41 both play a part in homodimerization by hydrogen bonding across the dimer interface with S287 and stabilizes the neighboring S-loop, whereas dephosphorylated Ser203 induces the fit of S-loop into the neighboring NAD-binding pocket by forming atomic interactions with three other S-loop residues. Among these residues, S50 and S287 were visible in the tunnel region, as illustrated in Figure 6B in red and cyan color, respectively. In addition, S-loop was depicted with yellow patches.

The tunnel region was further investigated for the amino acid sequence similarity between human and bacterium/parasite in order to guarantee that the proposed site incorporates distinctive features for identifying drug molecules that would specifically inhibit the enzyme of the infecting organism which is *S. aureus*. Colored based on sequence similarity between human and bacterium/parasite, the snapshots and the sequence alignment in Figure 6C clearly demonstrate the low degree of sequence conservation at the tunnel region. On the other hand, the structural RMSD value for the same region was exceptionally low at around 0.34 Å.

### *S. aureus* Pyruvate Kinase Displayed One Allosteric Site at the Center Cutting Across the Interface Region and Another at the Junction of A/C Domain

**FIGURE 7:**
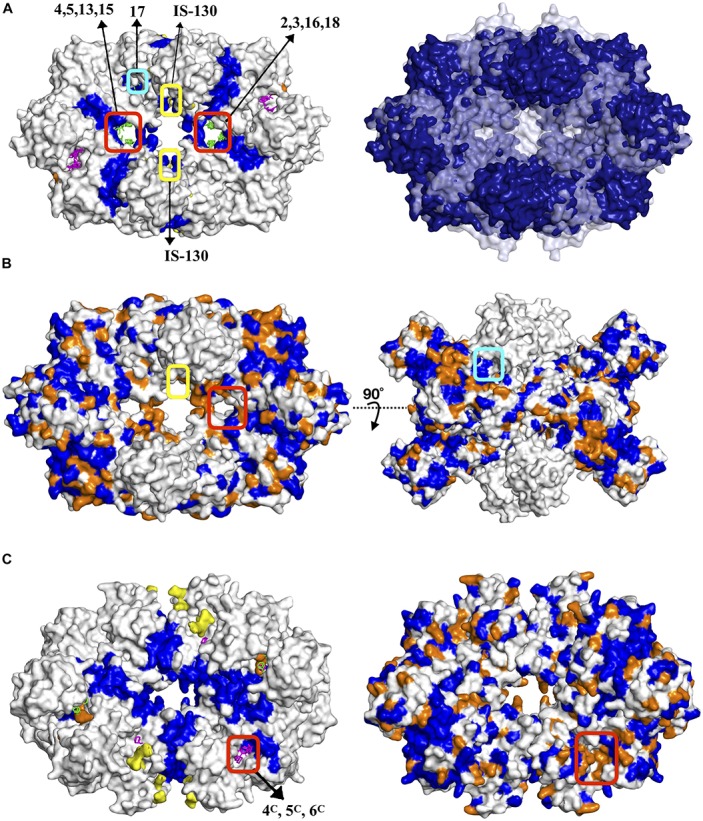
**(A)** Potential allosteric sites in *S. aureus* pyruvate kinase along with structural alignment of *S. aureus* and human PKs. Dark blue represents regions similar in both species, whereas white and pale blue regions correspond to unmatched regions, respectively. Sequence similarity illustrated on **(B)** a snapshot with two different views where proposed site encircled in red, well-reported IS–130 bound allosteric site encircled in yellow, proposed site with cluster ID 17 encircled in cyan. See caption of Figure 5 for color coding. **(C)** Top druggable site strongly proposed as a potential allosteric site in *L. mexicana* pyruvate kinase.

As listed in Table 3, the top druggable site corresponds to a region which is located at an opening in the center of the receptor and crossing an interface region. Its symmetric counterpart can also be observed at the other side of the orifice and both of these clusters were listed as top two druggable sites in Table 3. Moreover, a well-known allosteric site exists in the same orifice which accommodates the inhibitor IS-130 (N’-[(1E)-1-(1H-benzimidazol-2-yl)ethylidene]-5-bromo-2-hydroxybenzohydrazide) which was previously identified by Cilies and his coworkers as a potential allosteric inhibitor targeting methicillin-resistant Staphylococcus aureus (MRSA) (Axerio-Cilies et al., 2012). As illustrated in Figure 7A with yellow sticks at the top and bottom of the orifice, it is located at the so-called small C-C interface separating the subunits of the receptor, thus by disrupting the essential salt bridges which help to stabilize the small C-C interface and lock the tetramer in the active R-state, it may prevent the conformational transition to an active state (Morgan et al., 2010). Our newly proposed target site indicated by green sticks on the right and the left side of the orifice (encircled in red) is passing directly through the so-called large interface region, thus might eventually affect the rocking motion of the subunits necessary for activation. The human pyruvate kinase has a potential druggable site in the same corresponding region, however, the center of the receptor has a distinct shape with a nearly closed orifice almost inaccessible to the other side (see Figure 7A). Furthermore, this predicted druggable site coincide with the same pocket where quinolone sulfonamide activators bind (Kung et al., 2012). Besides, sequence alignment indicates this area with high amounts of variations which further emphasizes our proposed site as an ideal target for species-specific drug design (see Figures 7B and Supplementary Figure S4A). The remaining four clusters appeared in the vicinity of each of the four catalytic sites, as illustrated in Supplementary Figure S3, hence do not suggest an allosteric feature. A second alternative allosteric site in *Sa*PK appeared at the junction of A and C domain of one subunit as depicted by cluster with id 17 encircled with cyan in Figures 7A,B. No cluster was observed in human PK at the same corresponding site. Furthermore, sequence similarity analysis displayed a high degree of variation which indicated a likelihood of species specificity.

### *L. mexicana* Pyruvate Kinase Displayed a Distinct Allosteric Site Nearby an Interface Region

As illustrated in Figure 7C, a distinct druggable site on *Lm*PK was observed in the vicinity of an interface region, far away from both catalytic sites and the central region. Based on a low degree of sequence conservation, it is likely to provide a distinct binding site for specific drug candidates (see Supplementary Figure S4B). The remaining four druggable sites listed in Table 3 were detected at each of the four catalytic sites. Furthermore, there was no druggable site at the center which satisfied 50% frequency shift threshold as in human or bacteria. On the other hand, four isolated consensus sites have been detected at the center at the same exact locations as in human or bacteria, but in terms of effecting/shifting the frequency of the normal modes, they remained moderately within the range of 25–50%. Still, they can be proposed as possible target sites for species-specific drug design studies for *L. mexicana* PK.

### Critical Assessment of Binding Pockets With DoGSiteScorer

Our findings were compared to potential binding pockets predicted by the algorithm DogSiteScorer which is a grid-based method solely based on protein’s tertiary structure divided into subpockets, each assigned to a score value. DogSite scores appear between 0 and 1 with the most probable binding pockets displaying score values closer to 1. Each one of our druggable sites previously listed in Table 3 was re-evaluated based on the scores of DogSite pockets to which they overlapped. As shown in Table 4, high-score DogSite pockets coincided successfully with our predicted top druggable sites.

**TABLE 4:** Top druggable sites for each enzyme from each species and their corresponding DogSite binding pockets with score and rank.

Enzyme	*S. aureus*	Region	Score*/rank	Parasite	Score/rank	Region
PFK	4-5-7-8-12-15-17	Allosteric	0.81/3	1-9-13-6AB-7AB	0.80/4	Allosteric
	1-2-3-6-11-16	Allosteric	0.81/3	7-11-5CD-12CD	0.80/4	Allosteric
	2A-10A-11A	Catalytic	0.49/13	5-6CD-9CD-11CD	0.80/4	Allosteric
	2D-9D-11D	Catalytic	0.49/13	2A-3A-5A-8A	0.81/3	Catalytic
	2B-10B	Catalytic	0.78/6	3-12-13AB	0.80/4	Allosteric
				6A-7A	N/A	Catalytic
				10B-11B	0.53/16	Allosteric
				7D-10D	0.52/17	Allosteric
GADPH	1A-2A-5A-7A-8A-9A-2	Catalytic	0.80/2	1D-3D-4D-6D-7D-9D-3	0.48/6	Catalytic
	2B-3B-4B-7B-8B-5	Catalytic	0.76/3	2A-3A-4A-5A-7A-12	0.44/8	Catalytic
	3C-4C-6C-7-8-12	Catalytic	0.73/4	1B-6B-7B	0.72/3	Catalytic
	3D-4D-5D-3-14	Catalytic	0.64/8	2C-4C	0.81/1	Catalytic
	1-4-6-10-11	Allosteric	0.80/2			
PK	2-3-16-18	Allosteric	0.76/6	4C-5C-6C	0.42/9	Allosteric
	4-5-13-15	Allosteric	0.71/8	1B-9B-11B	0.81/4	Catalytic
	1A-2A-6A	Catalytic	0.58/18	9C-5-14	0.83/3	Catalytic
	1B-2B-8B	Catalytic	0.57/19	8-8A	0.81/4	Catalytic
	2C-3C-5C	Catalytic	0.63/16	7-13	0.81/4	Catalytic
	2D-3D-4D	Catalytic	0.53/21			

*Minimum-maximum range for score values:S. aureus PFK: [0.14-0.88]; T. brucei PFK:[0.16-0.86];S. aureus GADPH: [0.14-0.83]; T. cruzi GADPH:[0.15-0.81];S. aureus PK: [0.15-0.84]; L. mexicana PK:[0.11-0.86].

For *S. aureus* PFK, the predicted top druggable sites overlapped with the DogSite pocket ranked in third with 0.81 score value. The top two DogSite pockets with only slightly higher scores, 0.87 and 0.88, corresponded to catalytic regions where ATP binds (see Supplementary Figures S5A,B). The top druggable site in *T. brucei* which corresponded to the same location as in *S. aureus* and proposed to be allosteric, successfully coincided with a DogSite pocket of 0.8 score value which was the fourth highest. Interestingly, a second alternative allosteric site which was observed at the interface and proposed for *T. brucei* PFK as outlined with a yellow rectangle in Figure 6C, displayed a favorable DogSite pocket with 0.81 score value as also depicted in Supplementary Figure S5C. Moreover, the top score DogSite pocket in *T. brucei* PFK was detected in the interior region of the receptor unlike other binding cavities reported so far (see Supplementary Figure S5D).

For *S. aureus* GADPH, the tunnel region proposed to be an allosteric site displayed a pocket with 0.8 score value ranked in second (see Supplementary Figure S6A). Almost all catalytic regions overlapped with high-score pockets (see Supplementary Figures 6B,D). Interestingly, the corresponding tunnel region in *Cruzi* GADPH which did not appear among druggable sites due to its moderate frequency shift coincided with a favorable DogSite binding pocket with 0.80 score value ranked in second (see Supplementary Figure S6C). This finding increases the likelihood of the same tunnel region to be an allosteric site in parasite species as well, despite its relatively low frequency shift.

The new allosteric region proposed for *S. aureus* PK at the center of the structure neighboring the large interface coincided with the DogSite pocket ranked in sixth with a value of 0.76 which is not far from the highest score of 0.84 obtained for this structure (see Supplementary Figure S7A). On the other hand, catalytic regions appeared as druggable sites in our list were not strongly selected by DogSite. For *L. mexicana* PK, the proposed allosteric site located far from the origin and nearby an interface was not a highly favorable pocket for DogSite with only 0.42 score value ranked in the ninth position. On the other hand, the remaining four catalytic sites coincided well with highly scored DogSite pockets (see Supplementary Figure S7B).

### Support From AlloSigMA Server

Finally, our proposed allosteric sites were evaluated with AlloSigMA tool (Guarnera and Berezovsky, 2016b; Guarnera et al., 2017) which quantifies the allosteric effect of a ligand binding and/or mutation at a site on the basis of a per-residue free energy which is obtained by solving all possible protein local configurations. For our three allosteric enzymes, we investigated the effect of a ligand binding to our top druggable sites in *S. aureus* only. Other druggable sites and species will be considered in a future work.

**FIGURE 8:**
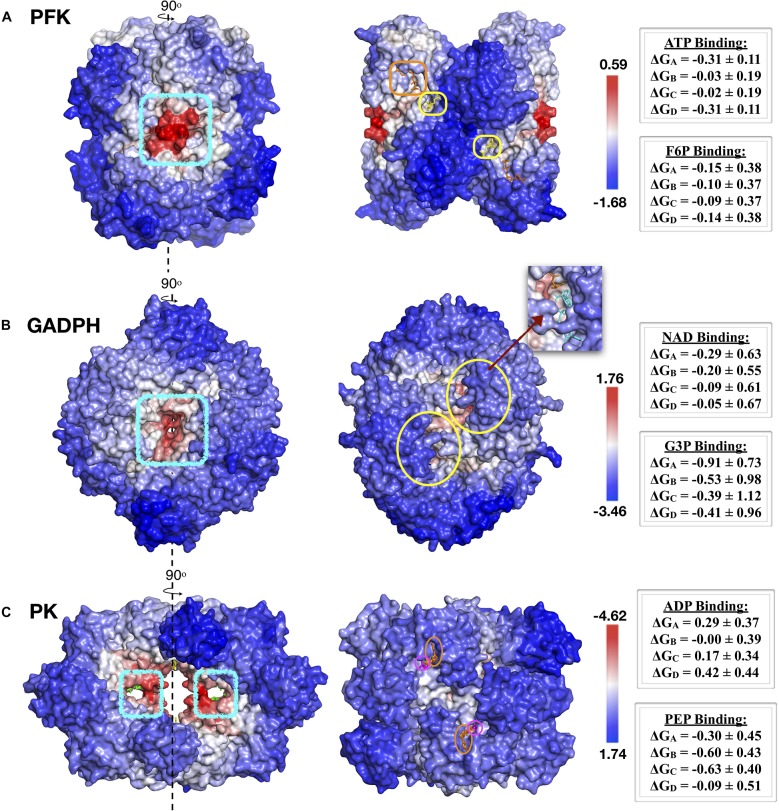
AlloSigMA results for top druggable sites and their symmetric counterparts in **(A)** PFK, **(B)** GADPH and **(C)** PK enzymes of *S. aureus* species. Proposed sites depicted with blue circles on the left, while catalytic regions indicated on the right side. Red and blue regions correspond to regions with decreased (ΔG < 0) and increased (ΔG > 0) dynamics, respectively.

Accordingly, the ligand binding to the proposed top druggable site and its symmetric counterpart in each of three enzymes caused a fair amount of decrease in residue dynamics in all catalytic regions. In phosphofructokinase, the highest decrease in allosteric effect was quantified by a negative mean free energy of –0.31 ± 0.11 and –0.15 ± 0.38 for ATP and F6P binding sites, respectively. Mean ΔG values of all four catalytic sites were listed as in Figure 8A. All four catalytic regions encircled in yellow for F6P displayed a comparable degree of mean ΔG which was around –0.1, whereas ATP binding site encircled in orange displayed two different values, one nearby –0.3 and the other –0.02.

Similarly analysis was conducted for the known allosteric site of *Sa*PFK illustrated in Figure 4, for comparison only. Binding of an effector molecule at the allosteric site is known to increase the activity of the receptor. Ligand binding with AlloSigMA exhibited a moderate amount of increase in the dynamics of F6P binding site with mean ΔG values varying between 0.12 and 0.25, whereas ATP binding site in two of the four monomeric units displayed a decrease in dynamics with a mean ΔG value of –0.5.

In GADPH, there exist four catalytic sites in which the substrate glyceraldehyde 3-phosphate (G3P) as well as the cofactor NAD binds. Two of these sites were illustrated with yellow circles as in Figure 8B, while the remaining two are on the opposite face of the receptor. The probe ligand was bound on both sides of the tunnel simultaneously. Accordingly, all four G3P sites displayed negative ΔG values between –0.66 ± 0.84 and –0.24 ± 1.1, whereas only two NAD binding sites showed unaltered dynamics with low positive ΔG values, 0.05 ± 0.67 and 0.09 ± 0.61. The proposed tunnel region clearly demonstrated a fair amount of allosteric effect on all four catalytic regions.

Finally, for pyruvate kinase, the allosteric effect via ligand binding to two symmetric proposed sites at the central region as depicted in Figure 8C, manifested itself as a moderate amount of decrease in the dynamics of each of the four catalytic regions where the substrate PEP would bind. On the other hand, all four ADP binding sites displayed only a slight increase in their dynamics. Furthermore, a similar analysis was conducted for the known allosteric site, which was occupied by the allosteric inhibitor IS-130 at the central region as illustrated in Figure 7A. Surprisingly, the allosteric effect was the opposite of that observed for our proposed site, with an increase in dynamics in the majority of PEP and ADP binding sites with mean ΔG values as high as 0.78 ± 0.21.

## Concluding Remarks

Our new approach consisting of a combination of well-established algorithms such as normal mode analysis using elastic network model and solvent-molecule binding site detection algorithm along with sequence and structural alignments demonstrated an exceptional prediction power for discovering alternative allosteric sites in the protein which were proposed as potential target sites for species-specific drug design efforts. The fact that nearly all well-reported catalytic and allosteric sites for three glycolytic enzymes have been identified undoubtedly supports the accuracy of our findings. Besides, several alternative allosteric sites have been identified for each one of three enzymes. *Sa*PFK presented a novel allosteric site which had one of the highest DogSiteScore value in addition to an allosteric effect perturbing the dynamics of all four catalytic regions. The second glycolytic enzyme, GADPH, presented the tunnel region as a potential allosteric site. Notably, this tunnel region incorporates the critical S-loop which owns the universal regulatory effect of the enzyme activity via its phosphorylation. The ligand binding to two symmetric sites at the tunnel region induced a fair amount of decrease in all four catalytic regions of the receptor. Finally, the two symmetric binding sites proposed for pyruvate kinase at the central region, exhibited allosteric features which were stronger than the known allosteric inhibitor sites nearby.

Although our current work was focused on allosteric enzymes only, the remaining seven glycolytic enzymes that do not display any allosteric feature in their functioning can be investigated using the same approach to identify potential allosteric sites that might be used to regulate the enzymatic activity of these enzymes. As our current strategy is solely based on the intrinsic nature of allostery supposedly owned by all proteins, there is always a likelihood of encountering a novel allosteric site that will be proposed as a target region for developing effective allosteric drugs.

## Data Availability Statement

All datasets generated for this study are included in article/Supplementary Material.

## Author Contributions

All authors listed have made a substantial, direct and intellectual contribution to the work, and approved it for publication.

## Conflict of Interest

The authors declare that the research was conducted in the absence of any commercial or financial relationships that could be construed as a potential conflict of interest.
