# Supplementary material for: Identification of Alternative Allosteric Sites in Glycolytic Enzymes for Potential Use as Species-Specific Drug Targets
Source: Front Mol Biosci. 2020 May 14;7:88. doi: 10.3389/fmolb.2020.00088 (PMC7240002; doi:10.3389/fmolb.2020.00088)
Supplement: Supplementary file 1 [file Data_Sheet_1.docx]

Supplementary Material

**Supplementary Table S1.** Distribution of consensus sites (CS) among druggable sites in phosphofructokinase (PFK)

| **Druggable Site ID** | **S.aureus**  **(PDB id: 5XZ7)** | **T. Brucei**  **(PDB id: 3F5M)** | **H. Sapiens**  **(PDB id: 4RH3)** |
| --- | --- | --- | --- |
| **1** | 1A***** – 6A***** | 2A – 3A – 5A – 8A | 1A – 3A****** – 9A****** – 11A |
| **2** | 2A – 8A****** – 10A –11A | 6A – 7A | 2A****** – 4A****** |
| **3** | 5A***** – 12A***** | 4B***** – 7B***** | 2B****** – 5B – 10B |
| **4** | 1B***** – 5B***** | 3B****** – 5B****** – 6B****** – 8B****** – 10****** | 3C****** – 4C – 8C |
| **5** | 2B – 10B | 1C***** – 3C****** – 6C****** | 1D****** – 5D – 6D – 7D |
| **6** | 6B* – 11B* | 4C***** – 5C****** – 7C***** | 6 – 11 |
| **7** | 1C***** – 6C***** | 2D****** – 3D****** – 6D****** | 3 – 5 – 9 |
| **8** | 2C – 11C****** | 4D***** – 5D****** | 10 – 12 |
| **9** | 5C***** – 10C***** | 2***** – 5 – 6CD – 9CD – 11CD | 1B****** – 4B – 9B |
| **10** | 1D***** – 7D***** | 3 – 12 – 13AB | 7B***** – 11B***** – 12B****** |
| **11** | 2D – 9D – 11D | 4****** – 7 – 11 – 5CD – 12CD | 2C****** – 6C – 9C |
| **12** | 1 – 2 – 3 – 6 – 11 – 16 | 1 – 9 – 13 – 6AB – 7AB | 2D – 4D****** |
| **13** | 4 – 5 – 7 – 8 – 12 – 15 - 17 | 10B – 11B | 6B – 7AB – 1 |
| **14** |  | 7D – 10D | 4 – 7 – 8 – 11CD |
| **15** |  |  | 2 – 5C |

*Clusters with FS < 25% are eliminated.

** Clusters with 25% < FS < 50% are eliminated.

Isolated CS (separated by more than 4 Å) observed in Suppl Fig 1 are not included in this table. They are identified as: 7AB, 4B, 4A, 9AB, 8AB, 10, 14, 7CD, 10AB, 13, 9, 11CD, 9CD, 4D, 4C, 8CD for *S. Aureus*, 9A, 9B, 8D, 6C, 8C, 9C for *T. Brucei*, and 9AB, 6A, 10CD, 13, 8D for *H. Sapiens*).

**Supplementary Table S2.** Distribution of consensus site (CS) among druggable sites in glyceraldehyde-3-phosphate-dehydrogenase (GADPH)

| **Druggable Site ID** | **S.Aureus**  **(PDB id: 3HQ4)** | **T. Cruzi**  **(PDB id: 3DMT)** | **H. Sapiens**  **(PDB id: 4WNI)** |
| --- | --- | --- | --- |
| **1** | 1A-2A-5A-7A-8A-9A-2 | 2A-3A-4A-5A-7A-12 | 2A-3A***-**4A-5A*****-6A******-7A*- 7 |
| **2** | 2B-3B-4B-6B******-7B-8B-5 | 1B-3B******-6B-7B-8B******-9******-15****** | 1B*****-2B-3B*****-4B-5B*****-6B******-7B* |
| **3** | 3C-4C-6C-7C******-7-8-12 | 2C-4C-5C******-6C******-7C******-13******-14****** | 1C*****-2C-3C******-4C*****-5C-6C*****-7C*****-8C******-9C***** |
| **4** | 3D-4D-5D-3-14 | 1D-3D-4D-6D-7D-8D******-9D-3 | 2D-3D*****-4D*****-5D******-6D*****-7D***-**8D******-8 |
| **5** | 1-4-6-10-11 | 1******-2******-4-5******-6******-7******-8******-10******-1****** | 1-2-3-4-5-6 |

*Clusters with FS < 25% are eliminated.

** Clusters with 25% < FS < 50% are eliminated.

**Supplementary Table S3.** Distribution of consensus site (CS) among druggable sites in pyruvate kinase (PK)

| **Druggable Site ID** | **S.aureus**  **(PDB id: 3T0T)** | **L.mexicana**  **(PDB id: 1PKL)** | **H. Sapiens**  **(PDB id: 4G1N)** |
| --- | --- | --- | --- |
| **1** | 1A – 2A – 6A | 4A* – 5A* – 6A* | 1A** – 4A* – 8A – 12A |
| **2** | 3A* – 5A* | 8 – 8A | 3A – 9A |
| **3** | 1B –2B – 8B | 1B – 9B – 11B | 5A* – 7A* |
| **4** | 3B* – 5B* – 7B* | 4B* – 5B* | 1B – 9B |
| **5** | 6B** – 9B** | 4C – 5C – 6C | 4B** – 7B** – 3B** |
| **6** | 2C – 3C – 5C | 9C – 5 – 14 | 1C – 5C – 10C** |
| **7** | 1D – 6D* | 4D* – 5D* – 6D* | 3C ** – 9C** |
| **8** | 2D – 3D – 4D | 1** – 10** | 4C* – 8C* |
| **9** | 8D* – 9D** | 2** – 15** | 2D** – 4D** |
| **10** | 1** – 2 – 3 – 16 –18 | 3** – 9** – 11** | 6D** – 7D – 9D |
| **11** | 4 – 5 – 11** – 13 – 15 | 4** – 6** | 1 – 3** – 7** – 8** – 9** – 10** |
| **12** | 7** – 12 | 7 – 13 | 2 -4 -5–6–11**-12** |

*Clusters with FS < 25% are eliminated.

** Clusters with 25% < FS < 50% are eliminated.

Isolated CS (separated by more than 4 Å) observed in Suppl Fig 3 are not included in this table. They are identified as: 8A, 10C, 12B, 17 for *S. Aureus*, 3A, 12, 7B, 7C, 9A, 9D, 2B, 10B for *L.mexicana*, and 3D, 6A, 7C, 8B, 10B, 10A 11A, 8D for *H. Sapiens*.

**Supplementary Table S4.** List of all residues observed in each druggable site given in Table 3 for phosphofructokinase (PFK).

| Druggable Site ID | *S. aureus* | Parasite | *H. sapiens* |
| --- | --- | --- | --- |
| 1 | **A:**N130**/**D136‑T143/L145/N146/W181/T259‑D262/V264‑A266/  R268/I288‑N291  **B:**F137‑T143/L145/N146/W181/T259‑D262/V264‑A266/R268/  I288/N291 | **A:**L84/A85/R237/Q242/N390/L393/C395/T397/L398/L401/M417/ N420‑Y422  **B:**R8‑S11/N230/H236‑T238/  F241/Q242/Q282/V285/R435/L437/Q442/L443/Q446 | **B:**D182/M183/A316‑D318/  I320‑A322/R324/L347/N348‑H351/N541/F548‑D553/L556/N557/Y589/M593/S679/F681/D682/F685/I722‑N726/W750 |
| 2 | **C:**N130/F137‑T143/L145/N146/W181/T259/G260D262/V264/  A266/R268/I288/N291  **D:**N130/F137‑T143/W181/T259‑D262/V264‑A266/R268/I288/  N290/N291 | **C:**R8‑S11/R64/N230/H236/  R237/T238/F241/Q242/F278/  Q282/R435/Q442/L443  **D:**L84/A86/R237/N390/L393/L401/N420-Y422 | **D:**S32‑G34/Y64/A96‑C98/  F101/R102/G126‑S130/G133/S173/G218‑C221/R310/Q368 |
| 3 | **A:**S9‑S13/M16/I100/G101‑S105/G126/T127‑I132/M171‑R173/  E224/R254 | **C:**L84‑A86/D237/T390/L393/L401/N420‑I423  **D:**L8‑S11/N230/H236/T238/  F241/Q242/F278/Q282/S432/  R435/Q442/L443 | **A:**M183/T187/D188/L191/  Y223/V227/S315/F317/N390/F548/D553/P680‑G686/K688/I722  **B:**F681/N684‑G686/K688/I689/  I722/V727 |
| 4 | **D:**S9‑S13/M16/I100/G101‑S105/G126‑I132/M171‑R173/R254 | **A:**A16‑R18/C105G107/C109/  R173/G174/G197‑R203/V224/  P225‑S233/M272‑S276/E325/S341  /N343/R383/I414/V429‑R435 | **A:**D182/M183/A316‑A322/R324/L347‑H351/G541/D547‑T554/  L556/N557/Y589/M593/F681/  D682/F685/W750 |
| 5 | **B:**S9‑S13/M16/G101‑S105/G126‑I132/M171‑R173/E224/R254 | **A:**T10/S11/R64/N230/H236‑T238/F241/Q242/Q282/R435/Q442/L443/Q446  **B:**R237/T238/Q242/N390/L393‑L398/M417/N420‑Y422 | **B:**N541‑V543/G545‑F548/C587/Y589/I722/S723/Q730/Q738/  P746/E748‑W751 |
| 6 |  | **A:**R173/K270‑M272/R274/E325/D339‑G350/D377/S379/  Y380/R383 | **C:**G419‑A423/M425/R481/A537‑S540/M583‑G585/L590/E639/  H671/Q674 |
| 7 |  | **B:** K270‑M272/R274/E325/  K345‑I349/D377/S379/  Y380/R383 | **A:** R565/Q568  **B:**L191‑R193/I195/E196/D199/  D553‑A555/N557/T558/Q674‑  P680/R683 |
| 8 |  | **D:**K270‑M272/R274/E325/K345‑I349/D377/S379/Y380/R383 | **A:**L191‑R193/I195/E196/D199/  T554/N557/T558/D561/G676‑  P680/R683  **B:**R565 |
| 9 |  |  | **B:**G419‑A423/M425/R481/A537‑S540/N542/M583‑G585/E639/  Q674 |
| 10 |  |  | **C:**N541‑V543/G545‑F548/C587/Y589/I722‑K724/Q730/Q738/  P746/E748-W751 |
| 11 |  |  | **B:**T183/T187/D188/L191/Y223/L224/V227/S315/F317/D553/  P680‑D682/N684‑G686/K688  **C:**F681/N684‑G686/K688/I689/  R692/I722/V727 |

**Supplementary Table S5.** List of all residues observed in each druggable site given in Table 3 for glyceraldehyde-3-phosphate-dehydrogenase (GADPH)

| Druggable Site ID | *S. aureus* | Parasite | *H. sapiens* |
| --- | --- | --- | --- |
| 1 | **A**:N8‑G14/N33‑D35/E95‑F99/T101/I119‑P122/A149‑T153/  T176/H178/Y180‑Q184/S210‑A213/A232/R234/A238/T239/Y314/N316/E317/Y320 | **D**:N8‑R15/D38/E109‑G112/  T115/I133‑P136/A164‑T168/  M190/T192/H194‑K200/S224‑A228/A231/S247‑R249/P253/  D254/Y333/N335‑W337/Y339 | **A**:T52/H53/A180/K181/L203‑I206/P236‑V240/S283‑N287  **B**:S51/T52/A180/N204‑I206/P236‑V240/S283/N287  **C**:T52/A180/K181/L203‑I206/P  236‑V240/S283‑N287  **D**:S51/T52/A180/K181/L203‑I206/P236‑V240/S283‑N287 |
| 2 | **B**:G9‑L16/E95‑F99/T101/I119‑P122/A149‑T153/T176/H178‑  Q184/S210‑A213/A232/R234/A238/T239/N316/E317/Y320 | **A**:G9‑G14/D38/E109‑G112/  S134‑P136/A164‑T168/T192/  H194/S195/T197‑P199/D210/  T225‑A228/S247‑R249/P253/  D254/Y333/N335‑W337/Y339 | **A**:R13‑G15/E97‑G100/T103/I121‑P124/A150‑C152/T182/N316/  E317/Y320 |
| 3 | **C**:G9‑G14/E95‑G98/T101/I119‑P122/A149‑T153/T176/H178‑D183/S210‑A213/A232/R234/A238/T239/N316/E317/Y320 | **B**:R12/I13/P136/S165‑T168/  M190/T192/S194‑Q200/D210/S224‑A228/S247‑R249/P253/  D254/N335/E336/Y333/Y339 | **B**:G10‑R16/E97‑V101/T103/I121‑A123/S151/A183/T184/Y320 |
| 4 | **D:**R12/I13/E95‑G98/T101/I119‑P122/A149‑T153/T176/H178/Y180‑D183/N209‑A213/A232/R234/A238‑T239/Y314‑E317/Y320 | **C**:G9‑G14/S110‑G112/S134/  P136/S165‑T167/M190/T192/  H194/Y196‑Q200/D210/S224‑A228/S247‑R249/P253/D254/  Y333/N335/E336 | **C**:G10‑R16/E97‑V101/T103/I121‑A123/A183/Y320 |
| 5 | **A**:D48‑R53/Y180/A203/E204/N205/P236‑A238  **B**:D48‑M50/A203‑N205/P236/T239/S281/D282/V284  **C**:A203/E204/P236  **D**:D48‑G52/A203/E204/P236/  S281‑V284/ |  | **D:**N9‑R16/E97‑V101/T103/S122‑P124/A150‑C152/T182‑T184/  N316/E317/Y320 |

**Supplementary Table S6.** List of all residues observed in each druggable site given in Table 3 for pyruvate kinase (PK)

| Druggable Site ID | *S. aureus* | Parasite | *H. sapiens* |
| --- | --- | --- | --- |
| 1 | **B:**K260/N299/Y302/D303/A337‑D339/Y340/K342/L343/D346  **C:**K260/R264/N267/N299/Y302/D303/D346/R347  **D:**Y302/Q338‑Y340/K342/L343 | **C:**R19/R22/I23/L40/I41‑  S46/V76‑I78/C420/T427/C428‑T434/V437‑S439 | **A:**F26/L27/H29/M30/L33/K311/  C326/N350/V352‑G355/A388‑H391/Q393-F395/E397  **B:**F26/L27/M30/G52/K311/N350/L353/D354/A388‑L394/E397 |
| 2 | **A:**K260/R264/N267/N299/Y302‑G304/A337‑Y340/K342/L343/D346/R347  **D:**K260/R264/N299/Y302/D303/  A337-A337‑Y340/K342/L343/D346 | **B:**G86‑R90/D145/G176‑  P180/C182/V184/L186/  F212/R214/E240‑Q243/  D264/L265/V267/E268 | **A:**N75/S77/H78/K115/P117‑R120/D178/K207‑P212/A214/  V216/F244/R246/E272/N273/  G295‑L297/I299/E300 |
| 3 | **A:**R32/N34/S36/D66‑R73/D125/K156‑L160/V165/L167/A190/S192/F193/  K219/E221/N222/D245/M246/V248/  E249 | **C:**S53/K85‑R90/G176‑C182/  V184/L186/F212/R214/E240  /N241/D264/V267/E268 | **B:**R73/N75/S77/H78/D113‑G116/  E118‑R120/Y175‑G179/K207‑  N210/F241/S243/F244/K270/E272/  A293/G295‑L297/I299 |
| 4 | **B:**R32/N34/S36/D66‑R73/D125/G157‑L160/V165/L167/A190/S192/F193/  E221/N222/D245/M246/V248/E249 | **A:**P87‑I89/V177‑C182/V184/L186/F212/R214/E240/  N241/D264/L265/V267/E268 | **C:**R73/N75‑S77/D113‑G116/E118/S243/F244/K270/E272/M291‑  L297/A327/T328/M360/S362 |
| 5 | **C:**R32/N34/S36/H37/D66‑R73/D125/K156‑L160/V165/L167/A190/S192/  F193/K219/E221/N222/D245/M246/  V248/E249 | **D:**P87‑I89/G176‑V184/L186/F212/R214/E240‑Q243/V267/E268 | **D:**A42‑I47/N70/D357/C358/P449/I450/A463‑P471**/**F502 |
| 6 | **D:**R32/N34/S36/D66‑R73/D125/K156‑L160/V165/L167/A190/S192/F193/  K219/E221/N222/G244‑M246/V248/  E249 |  |  |


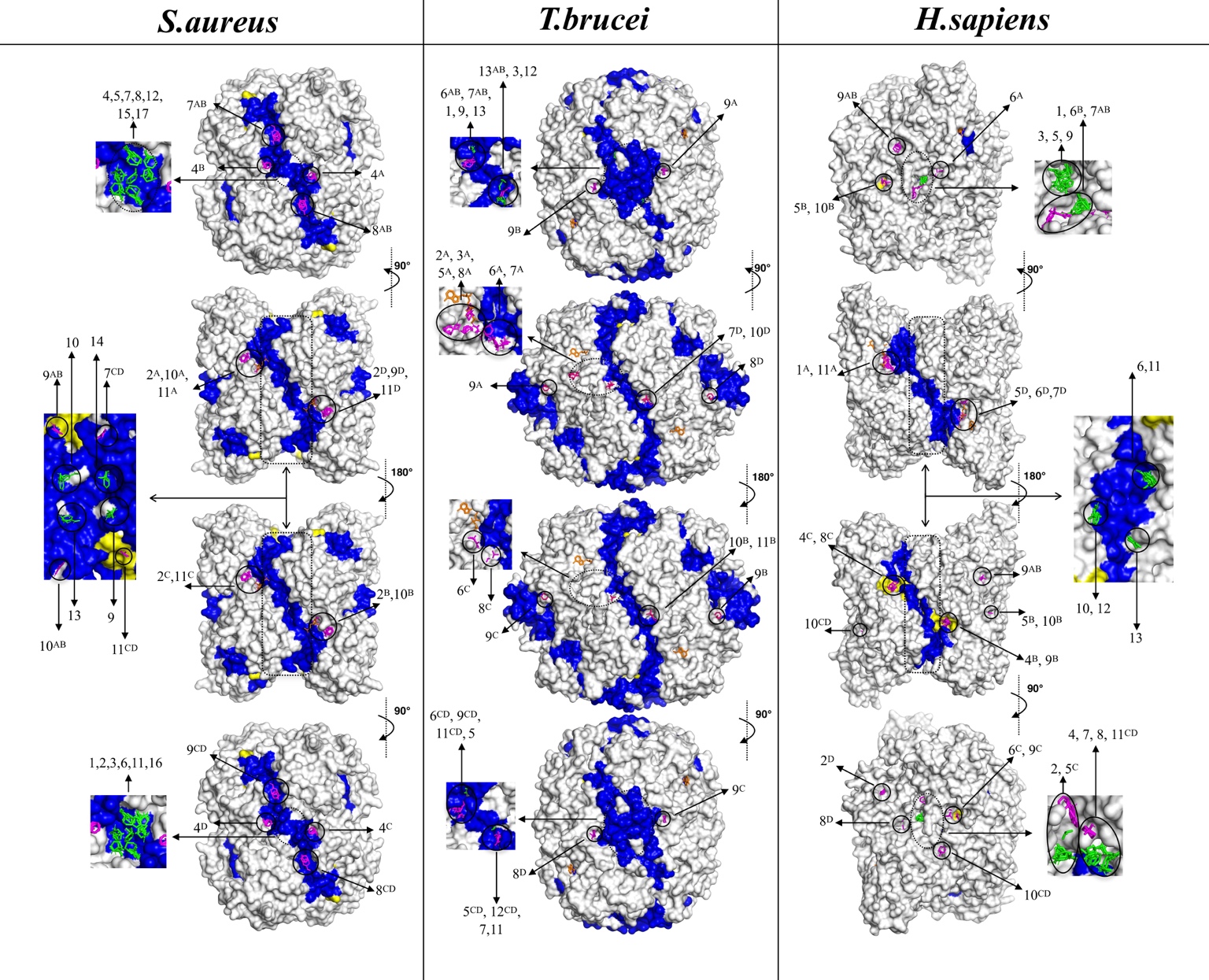


**Supplementary Figure S1.** Consensus sites listed as top druggable sites in Table 2 for phosphofructokinase in *S.Aureus*, *T.Brucei* and *H.Sapiens*.


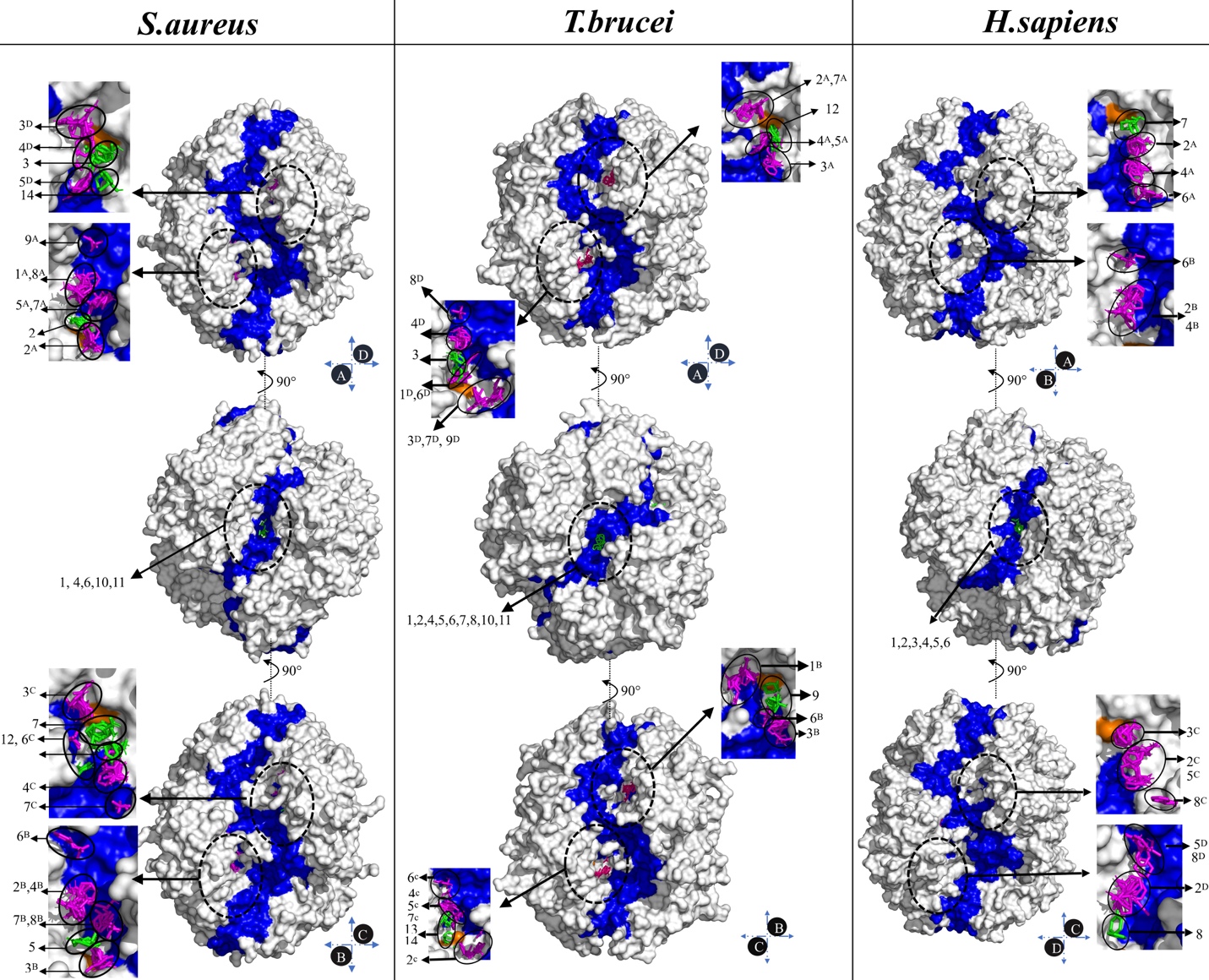


**Supplementary Figure S2.** Consensus sites listed as top druggable sites in Table 2 for glyceraldehyde 3-phosphate dehydrogenase in *S.Aureus*, *T.Brucei* and *H.Sapiens*.

**
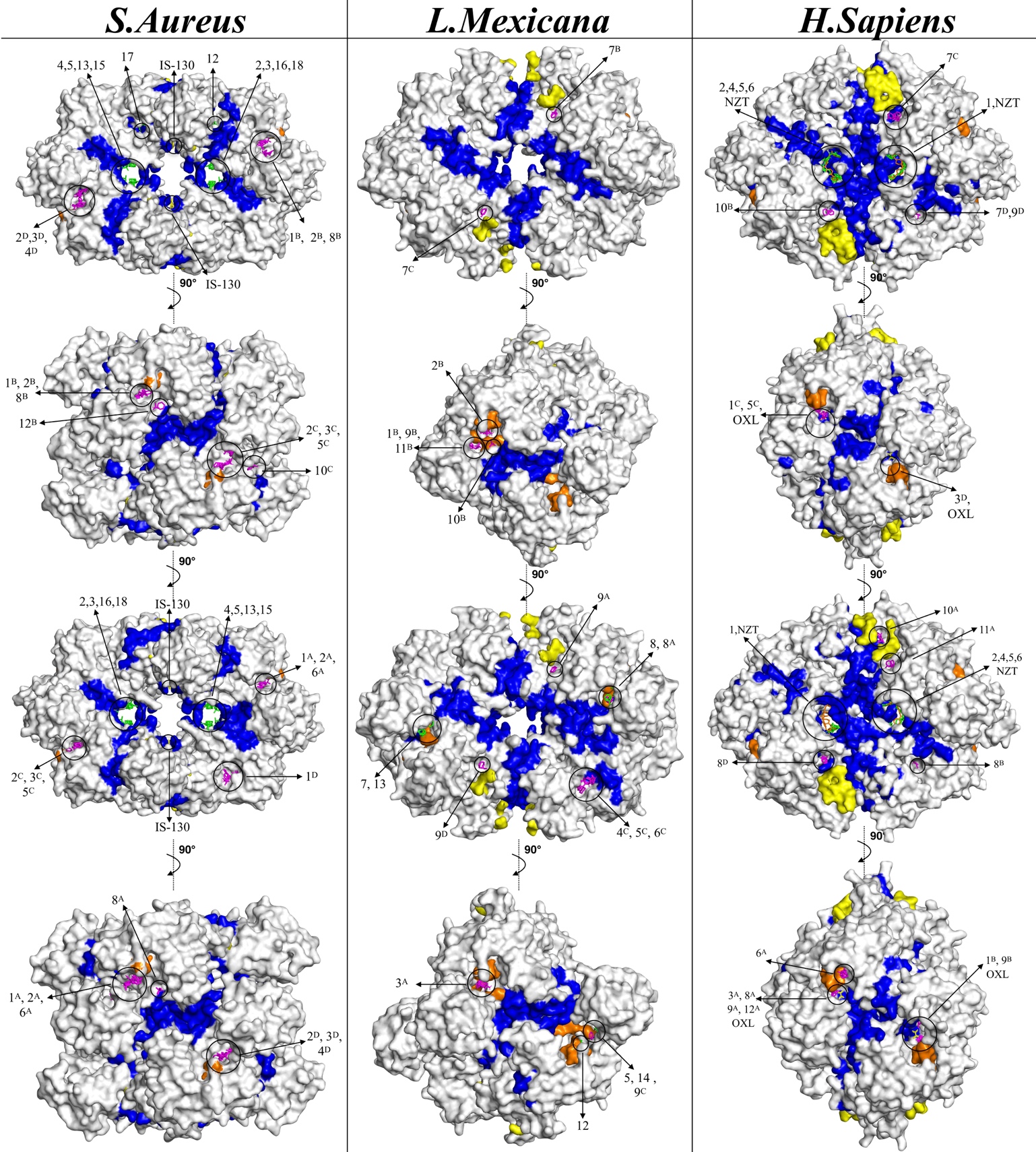
**

**Supplementary Figure S3.** Consensus sites listed as top druggable sites in Table 2 for pyruvate kinase in *S.Aureus*, *T.Brucei* and *H.Sapiens*.


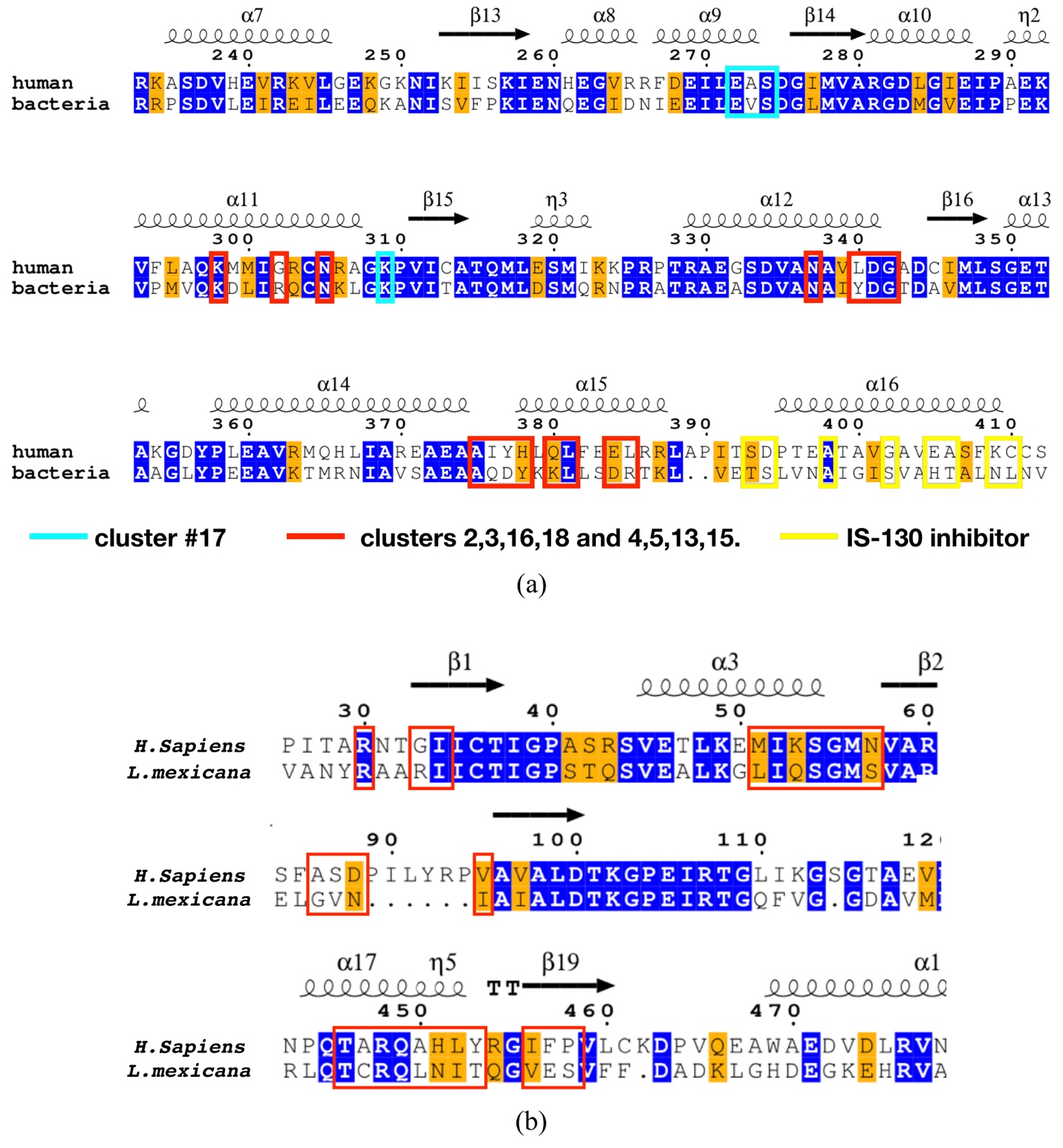


**Supplementary Figure S4.** Sequence alignment showing top druggable site sites encircled for a) human and *S.aureus* pyruvate kinase and b) human and *L.mexicana* pyruvate kinase. Similar, identical and dissimilar residues colored in orange, blue and white, respectively. ESPript 3.0 tool (Robert and Gouet 2014) used for graphical illustration.


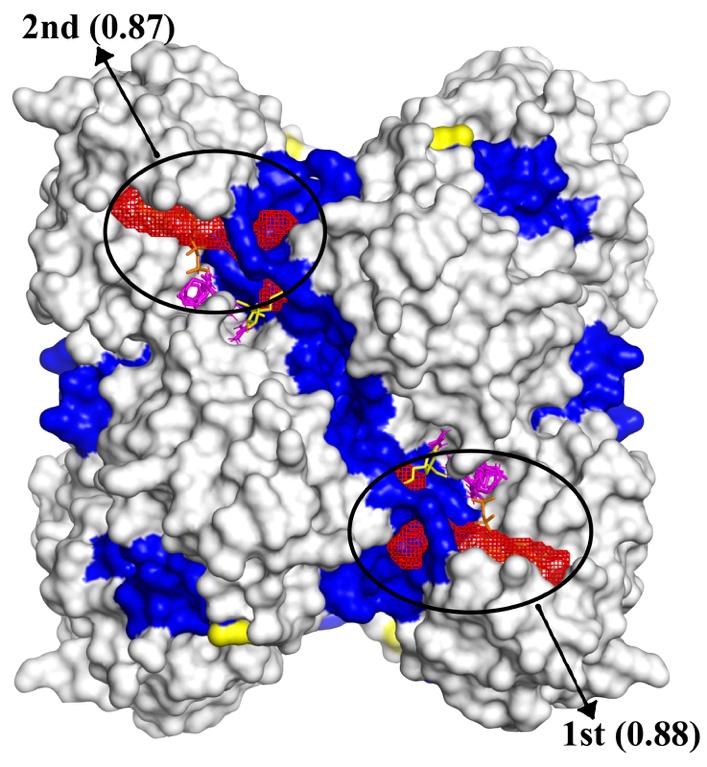

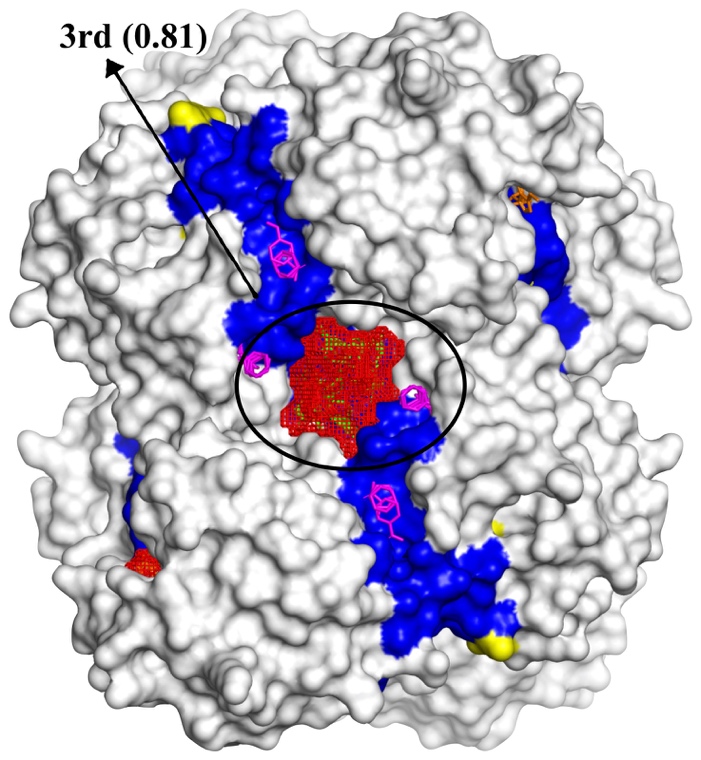


1. (b)


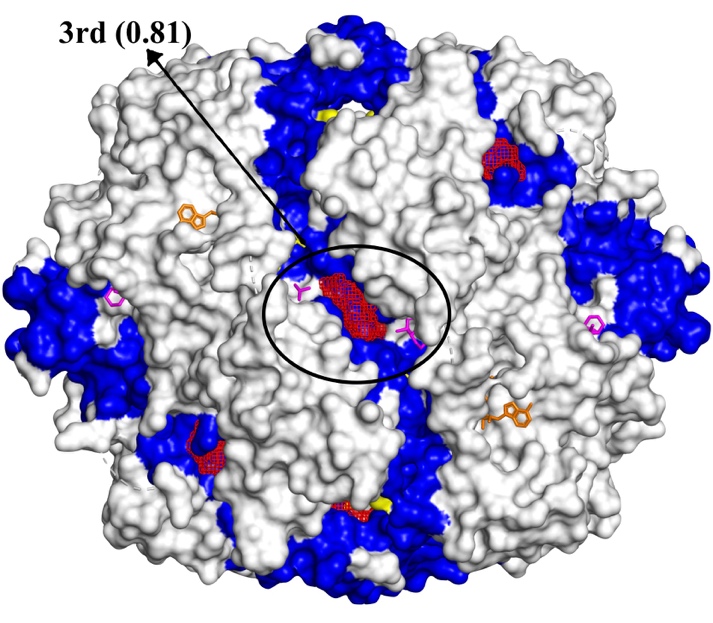

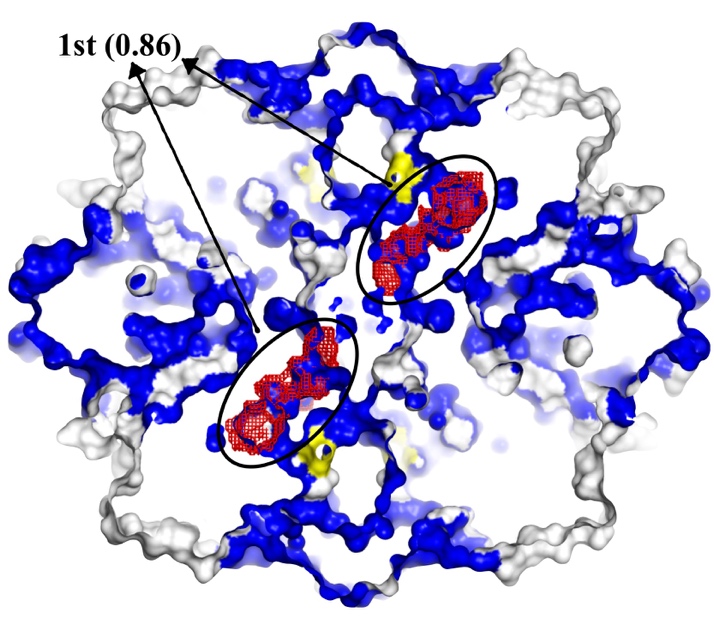


(c) (d)

**Supplementary Figure S5.** Snapshots showing the location of the binding pockets predicted by DoGSite in a),b) *S.aureus* phosphofructokinase and c),d) *T.brucei* phosphofructokinase.


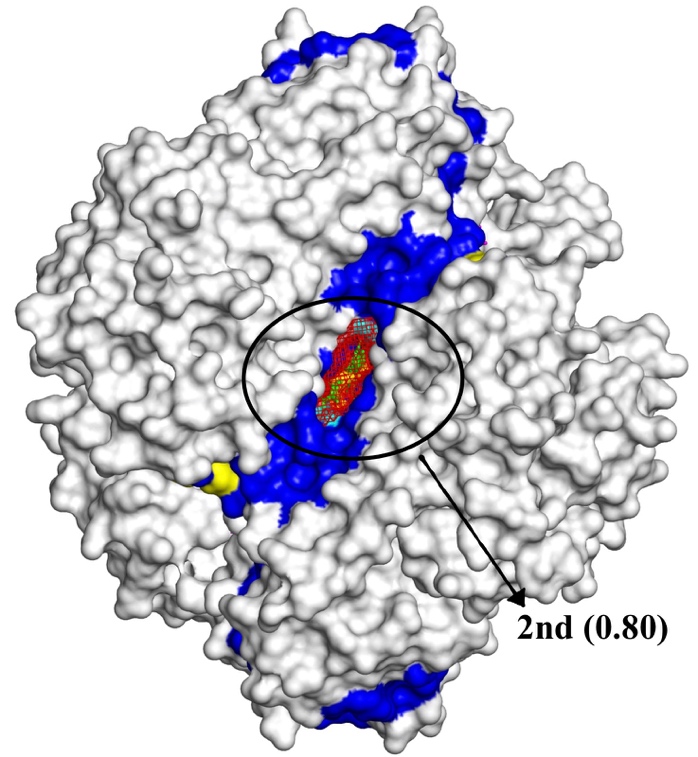

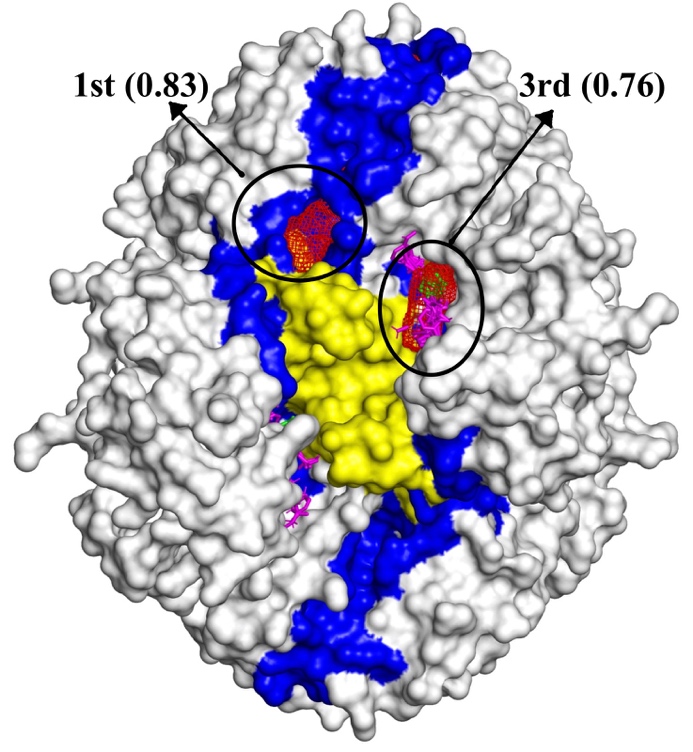


1. (b)


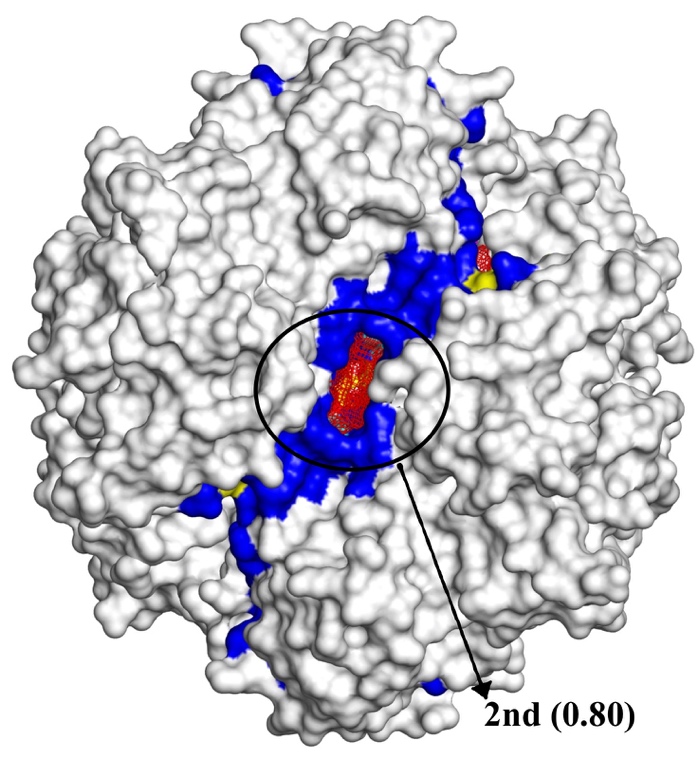

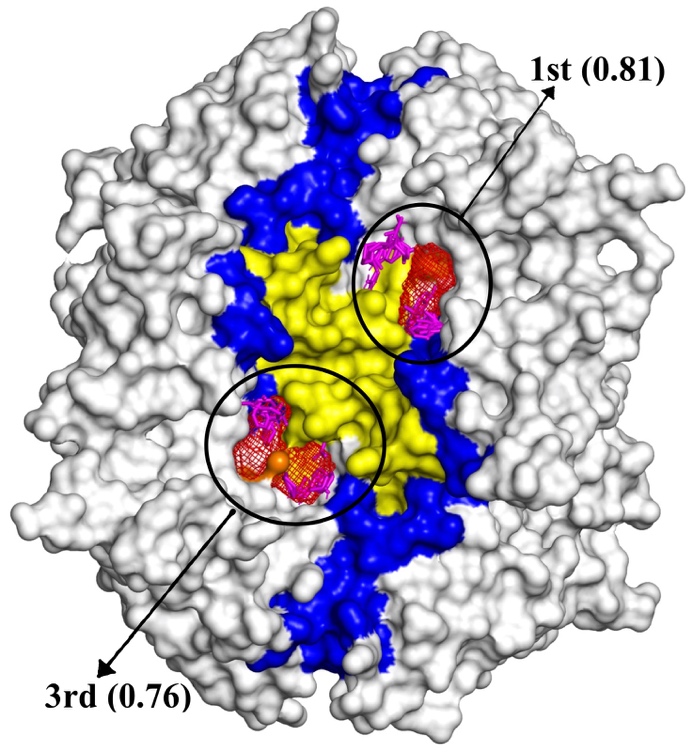


(c) (d)

**Supplementary Figure S6.** Snapshots showing the location of the binding pockets predicted by DoGSite in a),b) *S.*aureus glyceraldehyde 3-phosphate dehydrogenase and c),d) *T.cruzi* glyceraldehyde 3-phosphate dehydrogenase.


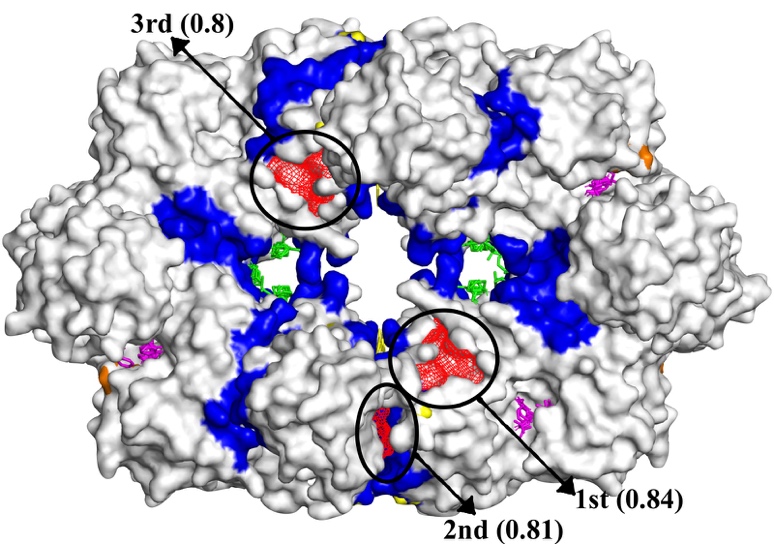


(a)


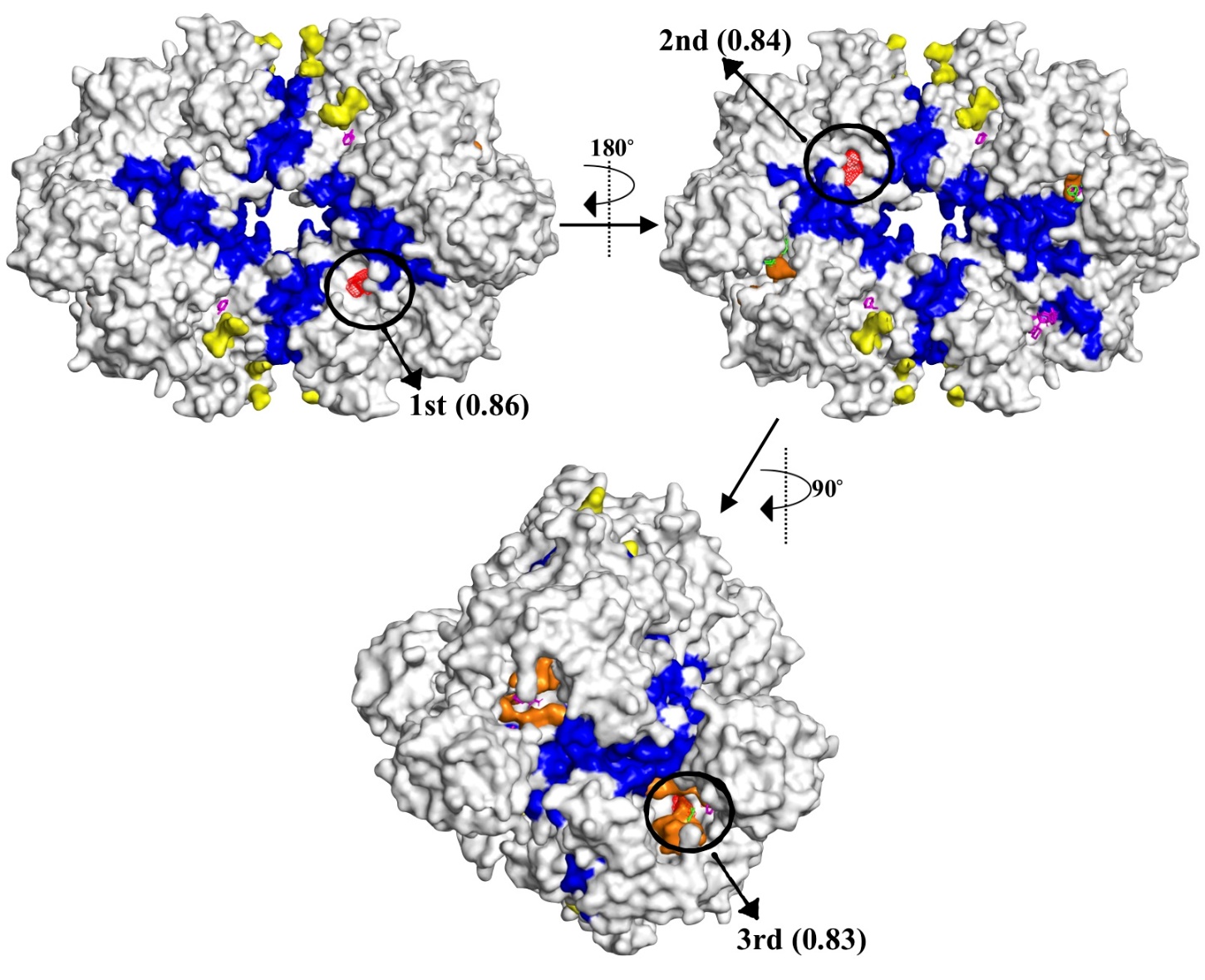


(b)

**Supplementary Figure S7.** Snapshots showing the location of the binding pockets predicted by DoGSite in a) *S.aureus* pyruvate kinase and b) *L.mexicana* pyruvate kinase.
